# The MYB transcription factor RcMYB1 plays a central role in rose anthocyanin biosynthesis

**DOI:** 10.1093/hr/uhad080

**Published:** 2023-04-21

**Authors:** Guoren He, Ren Zhang, Shenghang Jiang, Huanhuan Wang, Feng Ming

**Affiliations:** Shanghai Key Laboratory of Plant Molecular Sciences, College of Life Sciences, Shanghai Normal University, Shanghai, 200234, China; Shanghai Key Laboratory of Plant Molecular Sciences, College of Life Sciences, Shanghai Normal University, Shanghai, 200234, China; Shanghai Key Laboratory of Plant Molecular Sciences, College of Life Sciences, Shanghai Normal University, Shanghai, 200234, China; Shanghai Key Laboratory of Plant Molecular Sciences, College of Life Sciences, Shanghai Normal University, Shanghai, 200234, China; Shanghai Key Laboratory of Plant Molecular Sciences, College of Life Sciences, Shanghai Normal University, Shanghai, 200234, China

## Abstract

Rose (*Rosa hybrida*) is one of most famous ornamental plants in the world, and its commodity value largely depends on its flower color. However, the regulatory mechanism underlying rose flower color is still unclear. In this study, we found that a key R2R3-MYB transcription factor, RcMYB1, plays a central role in rose anthocyanin biosynthesis. Overexpression of *RcMYB1* significantly promoted anthocyanin accumulation in both white rose petals and tobacco leaves. In *35S*:*RcMYB1* transgenic lines, a significant accumulation of anthocyanins occurred in leaves and petioles. We further identified two MBW complexes (RcMYB1-RcBHLH42-RcTTG1; RcMYB1-RcEGL1-RcTTG1) associated with anthocyanin accumulation. Yeast one-hybrid and luciferase assays showed that RcMYB1 could active its own gene promoter and those of other EBGs (early anthocyanin biosynthesis genes) and LBGs (late anthocyanin biosynthesis genes). In addition, both of the MBW complexes enhanced the transcriptional activity of *RcMYB1* and LBGs. Interestingly, our results also indicate that RcMYB1 is involved in the metabolic regulation of carotenoids and volatile aroma. In summary, we found that RcMYB1 widely participates in the transcriptional regulation of ABGs (anthocyanin biosynthesis genes), indicative of its central role in the regulation of anthocyanin accumulation in rose. Our results provide a theoretical basis for the further improvement of the flower color trait in rose by breeding or genetic modification.

## Introduction

Anthocyanins are secondary metabolites of flavonoid biosynthetic pathways and are present in most flowering plants [1]. Anthocyanins not only give flowers and other organs rich colors, but also prevent damage from drought, ultraviolet radiation, low temperature stress, diseases, pests, and other adverse factors [2–5].

Anthocyanins range in color from orange-red (pelargonidin, cyanidin) to purple and pink-magenta (peonidin, malvidin, delphinidin, and petunidin) [6]. These pigments are produced by a branch of the phenylpropanoid metabolic pathway that is a flavonoid metabolic pathway. The genes for original phenylpropanoid biosynthesis include *phenylalanine ammonia lyase (PAL*), *cinnamate 4*-*hydroxylase* (*C4H*), and *4*-*coumarate*-*CoA ligase* (*4CL*). The anthocyanin early biosynthesis genes (EBGs) include *chalcone synthase* (*CHS*), *chalcone isomerase* (*CHI*), *flavanone 3*-*hydroxylase* (*F3H*), and *flavonoid 3*′-*hydroxylase* (*F3*′*H*), and the anthocyanin late biosynthesis genes (LBGs) include *dihydroflavonol 4*-*reductase* (*DFR*), *anthocyanidin synthase* (*ANS*), and *flavonoid 3*-*O*-*glucosyltransferase* (*UFGT*). The enzymes encoded by these genes work sequentially to generate specific anthocyanins [7].

The MYB transcription factors (TFs) are the most important transcription level regulatory genes of anthocyanins in the plant phenylpropane metabolism pathway. MYB-TFs can be divided into four groups according to the number of (1R, 2R, 3R, 4R) repeats and repeat sequence variation: 1R-MYB, R2R3-MYB, 3R-MYB, and 4R-MYB. In anthocyanin biosynthesis, some MYB-TFs function as activators (R2R3-MYB) and some function as repressors (R2R3-MYB and R3-MYB) [8]. For example, in sweet cherry (*Prunus avium*), PavMYB10.1 and PavMYB75 activate the cascade of anthocyanin downstream regulators and structural genes by increasing the expression level of anthocyanin biosynthesis genes (ABGs) [9]. A similar pattern of regulation has been reported for MYBs in peach (*Prunus persica*) (PpMYB7/10.1/10.4/9) [10], blueberry (*Vaccinium myrtillus*) (VmMYBA1 and VmMYBA2) [11], apple (*Malus domestica*) (MdMYB1/10/11 and MdMYBA) [12, 13], *Arabidopsis* (*Arabidopsis thaliana*) (AtMYB75/90) [14], eggplant (*Solanum melongena*) (SmMYB113) [15] and celery (*Apium graveolens*) (AgMYB1) [16]. The MYB-TFs that function as repressors of anthocyanin synthesis include *Fragaria* × *ananassa* FaMYB1 [17], *Arabidopsis* AtMYB4 [18], *Petunia hybrida* PhMYBx [19], peach PpMYB140 [19], PpMYB17–20 [21], *Populus tremula* PtrMYB182 [22], and apple MdMYB16 [23].

The MYB-TFs usually form MYB-bHLH-WD40 (MBW) complexes with bHLH (basic helix–loop–helix) and WDR (WD40-repeat) proteins, and these complexes can promote or inhibit anthocyanin biosynthesis [18]. The R3 repeats [with a conserved motif (D/E) LX2 (R-K) X3LX6LX3R] in MYB-TFs interact with the N-terminal MYB interacting region (MIR) of bHLHs, and together with WDR proteins form the MBW complex [18, 24]. In anthocyanin synthesis, there are usually multiple sets of MBW complexes that synergize or antagonize anthocyanin synthesis. In *Arabidopsis*, the four MYB-TFs PAP1–4 can form MBW complexes with bHLHs (TT8/GL3/EGL3) and WDR (TTG1) to further enhance anthocyanin biosynthesis synergistically [25]. In orchid (*Phalaenopsis equestris*), PeMYB2/11/12 independently activate the expression of *PeDFR*, and the presence of PebHLH1 further activates the expression of *PeDFR* and enhances red pigmentation [26]. When MdMYB10 was co-transformed with bHLHs in apple, the activity of *MdDFR* promoter was significantly enhanced. [13]. In woodland strawberry (*Fragaria vesca*), co-expression of *FvMYB10* and *FvbHLH33* can more strongly activate the promoters of *FvDFR* and *FvUFGT* to promote anthocyanin biosynthesis [27]. The small R3-MYB, which has a single R3 domain and lacks a putative activation domain, can inhibit MBW activity, and in general MYB inhibitors can competitively bind bHLH to reduce the activity of the MBW complex and decrease anthocyanin synthesis [28]. For example, IbMYB44 in *Ipomoea batatas* inhibits its own transcriptional activity by competitively binding bHLH2 in the MBW (MYB340-bHLH2-NaC56) complex, thereby inhibiting anthocyanin biosynthesis [29]. The MYB inhibitor MYBL2 can competitively bind to bHLHs (MYC1/EGL3/GL3/TT8), and these interactions can disrupt the MBW complex or the binding of the complex to the promoters of ABGs, resulting in decreased anthocyanin accumulation [30].

Only a few studies have reported that MYB-TFs may be involved in the anthocyanin biosynthesis of rose. RrMYB113 and RhMYB10 were supposed to play a role in anthocyanin synthesis [31, 32]. *RrMYB5* and *RrMYB10* are induced by wounding, and their expression leads to increased accumulation of anthocyanins and proanthocyanidins [33]. Therefore, the MYB-TFs and the MBW complex regulating flower color in roses have not been studied in depth.

Previously, we performed a detailed analysis of *R. hybrid* ‘Rhapsody in Blue’ petal RNA-seq data (accession number: PRJNA885821) and identified genes involved in rose petal color regulation. We noticed that the FPKM value of *RcMYB1* increased during flower coloration, suggesting thatzit may promote anthocyanin accumulation in rose flowers. Here, we analysed the regulation of RcMYB1 and two MBW complexes on rose pigment formation to explore the molecular regulation mechanism of rose color formation. These results provide new information for the regulation of rose color and will be useful for breeding roses and possibly other flowering plants with a wider range of flower colors.

## Results

### RcMYB1 *encodes an SG6 R2R3 MYB TF*

A phylogenetic tree of *RcMYB1* and other members of the MYB transcription factor family in *Arabidopsis* and some Rosaceae species was constructed. In the tree, RcMYB1 was in subgroup 6 (SG6), whose members are known to promote anthocyanin accumulation in plants (Fig. S1, see online supplementary material). In addition, *RcMYB1* showed high homology with *RrMYB113* of rugosa rose (*Rosa rugosa*), *RiMYB10* of red raspberry (*Rubus idaeus*), *FaMYB10* of strawberry (*F*. × *ananassa*), and *FvMYB10* of woodland strawberry (*F. vesca*) (Fig. S1, see online supplementary material). Multiple sequence alignment showed that *RcMYB1* contained the typical bHLH interaction motif, the ANDV motif, and the conserved R2R3 MYB DNA-binding domain [R/K]Px[P/A/R]xx[F/Y] motif (Fig. S2, see online supplementary material). The results indicate that RcMYB1 may be an activator of anthocyanin biosynthesis in rose.

**Figure 1:**
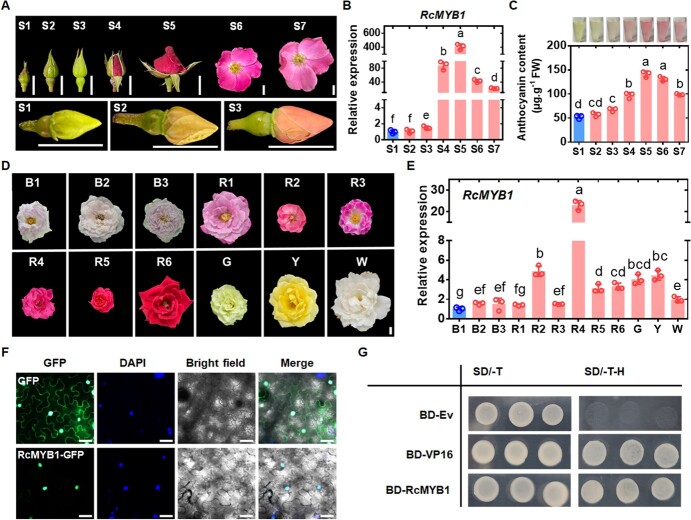
Identification of the transcription factor *RcMYB1*. **A** Seven stages of of *Rosa chinensis* ‘Old Blush’ development (S1, S2, S3, S4, S5, S6, and S7). Images were digitally extracted for comparison. The scale bar is 1 cm. **B** Relative transcript levels of *RcMYB1* in rose flowers at seven stages of development. **C** Anthocyanin content in petals of rose flowers at seven stages of development. **D** Differently colored rose flowers. B stands for blue-purple rose varieties: B1 is *Rosa hybrida* ‘Lavender Bouquet’; B2 is *R. hybrida* ‘Libellula’; B3 is *R. hybrida* ‘Kong Meng’. R stands for red rose varieties: R1 is *R. hybrida* ‘Muriel Robin’; R2 is *R. hybrida* ‘Angela’; R3 is *R. hybrida* ‘Yan Li’; R4 is *R. hybrida* ‘Ren Yue’; R5 is *R. hybrida* ‘Cherry Bonica’; R6 is *R. hybrida* ‘Black Magic’. G stands for green rose, and G is *R. hybrida* ‘Duo Lei’. Y stands for yellow rose, Y is *R. hybrida* ‘Yellow Leisure Liness’. W stands for white rose, W is *R. hybrida* ‘Gabriel’. Images were digitally extracted for comparison. The scale bar is 1 cm. **E** Relative transcript levels of *RcMYB1* in differently colored rose flowers. **F** Subcellular localization of RcMYB1-GFP transiently expressed in *Nicotiana benthamiana* leaf cells. Note that GFP fluorescence overlaps with that of DAPI (nuclear marker). The scale bar is 50 μm. **G** Transcriptional activation associated with RcMYB1 in yeast cells. SD/−T, SD-Leu-Trp medium; SD-T-H, SD-Trp-His medium. Values are means ± SDs (*n* = 3). Lowercase letters (a–f) in **B**, (a–d) in **C**, and (a–g) in **F** indicate significantly different values (Student’s *t* test, *P* < 0.05).

To study the transcript profile of *RcMYB1* during petal coloration of rose, we selected the petals of seven developmental stages of ‘Old Blush’ flowers (Figs S1–S7, see online supplementary material) (Fig. 1A) and determined the *RcMYB1* transcript levels and anthocyanin contents. The transcript levels of *RcMYB1* gradually increased as anthocyanins accumulated, with the highest transcript level at S5, and then gradually decreased as the anthocyanins degraded (Fig. 1B). Thus, the transcriptional profile of *RcMYB1* was consistent with the trends in anthocyanin accumulation during Figs S1–S7, see online supplementary material (Fig. 1C).

Next, we determined the transcript levels of *RcMYB1* in rose flowers with different colors, including blue-purple, red, green, yellow, and white (Fig. 1D). Interestingly, although *RcMYB1* showed relatively high transcript levels in red roses, they were equally high in green, yellow, and white roses, but low in several blue-purple roses (Fig. 1E). The yellow flower is conferred by carotenoids, and the white rose studied here had a strong fragrance. Thus, we speculate that *RcMYB1* may also be involved in the biosynthesis of volatile aromatic compounds and carotenoids.

To determine where RcMYB1 functions in cells and whether it has transcriptional activation activity, we performed a subcellular localization analysis and a trans-acting activity assay using a yeast system. In subcellular localization analysis, the RcMYB1-GFP signal was localized in nucleus cells of *Nicotiana benthamiana* leaves, consistent with its role as transcription factor (Fig. 1F). In the yeast system, RcMYB1-BD yeast cells and positive control VP16-BD also grew well on selective medium (SD/−Trp/-His), while negative control (BD) could not grow on selective medium (Fig. 1G). These results provided further evidence that *RcMYB1* functions as an activating transcription factor in the nuclei.

### RcMYB1 *promotes anthocyanin accumulation in rose petals and tobacco (Nicotiana tabacum) leaves*

**Figure 2:**
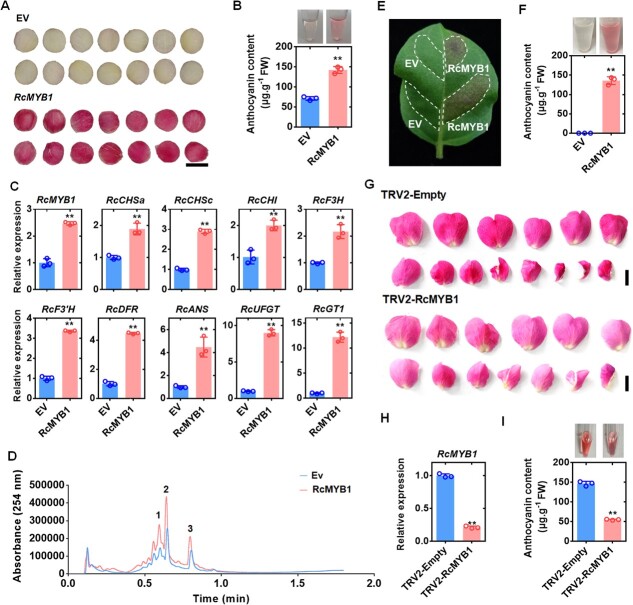
Transient overexpression of *RcMYB1* in rose petals and tobacco (*Nicotiana tabacum*) leaves. **A** Rose petals transformed with empty vector (EV) or vector containing *RcMYB1*. Images were digitally extracted for comparison. The scale bar is 1 cm. **B** Anthocyanin contents in rose petals expressing EV or *RcMYB1*. **C** Relative transcript levels of *RcMYB1* and anthocyanin-related genes in rose petals expressing EV or *RcMYB1*. **D** LCMS analysis of the anthocyanin composition after overexpression of *RcMYB1*: 1 and 3 indicate cyanidin derivatives, 2 indicate cyanidin. **E** Tobacco leaf transformed with EV or RcMYB1. White dotted lines show infiltrated zones. **F** Anthocyanin content in parts of tobacco leaf transformed with EV or *RcMYB1*. **G** The phenotype of rose petals after silencing *RcMYB1*. The scale bar is 2 cm. **H** The relative expression of *RcMYB1* in rose petals after silencing *RcMYB1*. **I** Anthocyanin contents in rose petals after silencing *RcMYB1*. Values are means ± SDs (*n* = 3). Asterisks in B, C, F, H, and I indicate significantly different values (Student’s *t* test, ^**^P < 0.01).

To clarify the function of *RcMYB1* in anthocyanin accumulation, we performed transient overexpression experiments in *Rosa hybrida* ‘Gabriel’ petals and tobacco (*N. tabacum*) leaves. At 5 d after transformation, the white petals in the control (harboring the empty vector, EV) showed a slight red color, while those transiently overexpressing *RcMYB1* had changed to deep red (Fig. 2A) and their anthocyanin content was significantly increased (Fig. 2B). The groups overexpressing *RcMYB1* also showed significantly increased transcript levels of ABGs (*RcCHSa*, *RcCHSc*, *RcCHI*, *RcF3H*, *RcF3′H*, *RcDFR*, *RcANS*, *RcUFGT*, and *RcGT1*) (Fig. 2C). To determine the major anthocyanin components in red petals upon *RcMYB1* overexpression, we examined the anthocyanin composition by liquid chromatography–mass spectrometry (LC–MS). The red color was mainly due to the increased contents of cyanidin and cyanidin derivatives (Fig. 2D). In tobacco leaves, the leaf areas injected with *35S:RcMYB1* showed a red color and accumulated anthocyanins (Fig. 2E and F). Further, we used virus-induced gene silencing (VIGS) to silence *RcMYB1* in ‘Old Blush’. Our results showed that petal color was significantly lightened and anthocyanin content was significantly reduced after the silencing of *RcMYB1* (Fig. 2G, H, I).

### Significant accumulation of anthocyanins in RcMYB1 transgenic lines in rose

**Figure 3:**
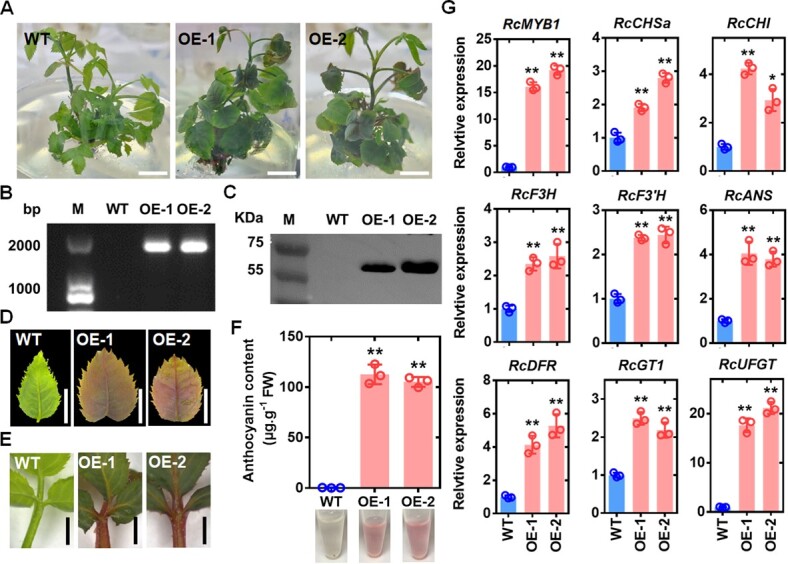
Effects of overexpression of *RcMYB1* in transgenic rose plants. **A** Phenotypes of wild type (WT) and two *RcMYB1*-overexpressing lines (OE-1 and OE-2). The scale bar is 1 cm. **B** Confirmation of OE lines by PCR amplification. **C** Confirmation of OE lines by western blot analysis with GFP antibody. **D** WT and OE lines leaf phenotypes. The scale bar is 1 cm. **E** Phenotypes of petioles of WT and OE lines. The scale bar is 1 cm. **F** Anthocyanin content in WT and OE lines. **G** Relative transcript levels of *RcMYB1* and anthocyanin-related genes in WT and OE lines. Values are means ± SDs (*n* = 3). Asterisks in **F** and **G** indicate significantly different values (Student’s *t* test, ^*^P < 0.05 and ^**^P < 0.01).

To further clarify the function of *RcMYB1* in rose anthocyanin biosynthesis, we transferred the full-length coding sequence of *RcMYB1* into the pCAMBIA2300 vector to obtain *35S:RcMYB1* transgenic rose lines (OE-1 and OE-2) (Fig. 3A). The rose transgenic lines were confirmed by amplifying partial sequences of the pCAMBIA2300 vector with *RcMYB1* by polymerase chain reaction (PCR) from genomic DNA, as well as by western blotting with an anti-GFP antibody (Fig. 3B and C). In the *RcMYB1*-OE lines, the leaves, petioles and stems significantly accumulated anthocyanins (Fig. 3D, E, F; Fig. S3, see online supplementary material). Further RT-qPCR analysis revealed up-regulation of *RcMYB1* and ABGs (Fig. 3G), consistent with the results of the transient expression experiments. These results suggested that RcMYB1 has extensive involvement in the regulation of anthocyanin biosynthesis to mediate anthocyanin accumulation.

### RcMYB1 physically interacts with RcTTG1 and RcbHLH42 or RcEGL1 to form MBW complexes

The MBW complex plays a core role in regulating anthocyanin biosynthesis. Therefore, we further identified the possible MBW complexes related to anthocyanin biosynthesis in rose. The phylogenetic tree was constructed using rose bHLH family proteins and reported bHLH proteins (AtMYC1, AtEGL3, AtGL3, AtTT8) related to anthocyanin biosynthesis in *Arabidopsis*. The tree showed that *RcbHLH42* and *RcEGL1* were closely related to these four bHLH proteins in *Arabidopsis* (Fig. S4, see online supplementary material). Another phylogenetic tree was constructed using *RcbHLH42*, *RcEGL1* and other reported bHLH proteins related to anthocyanin biosynthesis in other species. That tree showed that *RcbHLH42* and *RcEGL1* were closely related to *bHLH3* and *bHLH33* in strawberry, respectively (Fig. S5A, see online supplementary material). Multiple sequence alignment analysis revealed conserved bHLH motifs in RcbHLH42 and RcEGL1 (Fig. S6, see online supplementary material). According to the amino acid sequence of AtTTG1 in *Arabidopsis*, we selected the WDR gene, *RcTTG1*, with the highest homology from rose. A phylogenetic tree was constructed with the TTG1 proteins related to anthocyanin biosynthesis reported for different plant species and showed that *RcTTG1* was closely related to *FaTTG1* in strawberry (Fig. S5B, see online supplementary material). Multiple sequence alignment analysis showed that RcTTG1 contained a conserved tandem repeat WD motif (Fig. S7, see online supplementary material).

To further determine whether *RcbHLH42*, *RcEGL1*, and *RcTTG1* are related to anthocyanin biosynthesis, we examined their transcript levels in flowers at different stages of development. The transcript levels of *RcbHLH42* and *RcTTG1* increased during the early stage of anthocyanin accumulation, while *RcEGL1* showed a decreasing trend (Fig. S5C, see online supplementary material). Nevertheless, we found that the transcript levels of these three genes were significantly elevated in the transgenic lines (Fig. S5D).

To determine where RcbHLH42, RcEGL1, and RcTTG1 function in the cells, we performed subcellular localization. The results showed that the GFP signal of RcEGL1-GFP fusion protein was localized in the nucleus, while RcbHLH42-GFP and RcTTG1-GFP fusion proteins were localized in the nucleus and cytoplasm of *N. benthamiana* epidermal cells (Fig. S5E, see online supplementary material).

**Figure 4:**
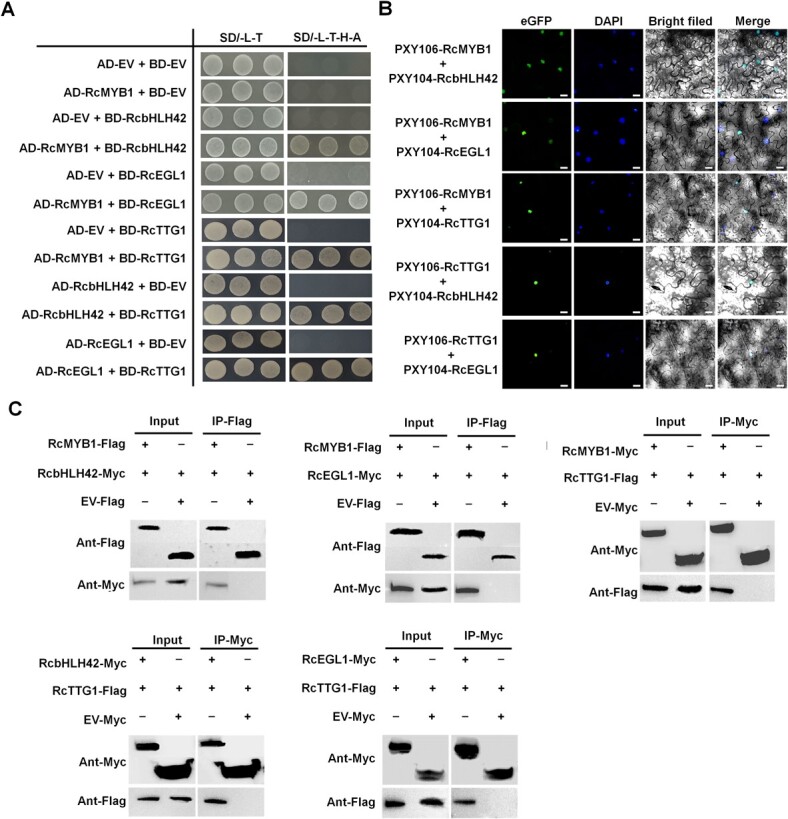
Analyses of potential of RcMYB1 to form MBW complexes with RcTTG1, RcbHLH42, or RcEGL1. **A** Yeast two-hybrid analyses demonstrating interaction between RcMYB1 and other components of MBW complexes. BD, DNA-binding domain; AD, activation domain; SD/−L-T, SD-Leu-Trp medium; SD-L-T-H-A medium SD-Leu-Trp-His-Ade medium. **B** Bimolecular fluorescence complementation analyses of demonstrating interaction between RcMYB1 and other components of MBW complexes in *N. benthamiana* leaves. Nuclei were counterstained with DAPI. Scale bar is 25 μm. **C** Coimmunoprecipitation assays demonstrating interactions among RcMYB1, two bHLHs (RcbHLH42 and RcEGL1), and RcTTG1.

To determine whether RcMYB1 could form an MBW complex with RcTTG1, RcbHLH42, or RcEGL1, we performed yeast two-hybrid assays (Y2H) assays. Directed Y2H assays validated that RcMYB1 interacted with the two bHLH proteins (RcbHLH42 and RcEGL1) and the WDR protein RcTTG1 (Fig. 4A), and RcTTG1 also interacted with the two bHLH proteins (RcbHLH42 and RcEGL1).

To verify whether RcMYB1, the two bHLHs (RcbHLH42 or RcEGL1) and RcTTG1 HAT1 interact with each other *in vivo*, we conducted bimolecular fluorescence complementation (BiFC) assays. When RcMYB1-nYFP was coinfiltrated with RcbHLH42-cYFP or RcbEGL1-cYFP or RcTTG1-cYFP, and when RcTTG1-nYFP was coinfiltrated with RcbHLH42-cYFP or RcbEGL1-cYFP into *N. benthamiana* leaves, the strong YFP fluorescence was located in the nucleus (Fig. 4B). The interactions between RcMYB1 and RcbHLH42 or RcEGL1 or RcTTG1, and between RcTTG1 and RcbHLH42 or RcEGL1 were also confirmed by Co-IP assays (Fig. 4C). These results indicated that RcMYB1, two bHLHs (RcbHLH42 and RcEGL1) and RcTTG1 can interact with each other to form MBW complexes.

### Two MBW complexes positively regulate anthocyanin accumulation in a functionally redundant manner

To determine whether RcbHLHs (RcbHLH42, RcEGL1), RcTTG1, and the two MBW complexes were involved in anthocyanin accumulation, we performed transient expression and transgenic assays in tobacco. Transient expression of *RcbHLH*s (*RcbHLH42*, *RcEGL1*) and *RcTTG1* alone did not promote anthocyanin accumulation in *N. tabacum* leaves (Fig. S8A and B, see online supplementary material), while tobacco leaves co-infected with genes encoding components of two MBW complexes, *RcMYB1*, *RcbHLH42* and *RcTTG1* (MBT) and *RcMYB1*, *RcEGL1* and *RcTTG1* (MET) accumulated more anthocyanins compared with those transiently expressing *RcMYB1* alone. There was no significant difference in anthocyanin accumulation between two lines containing different MBW complexes (Fig. S8A and B, see online supplementary material).

**Figure 5:**
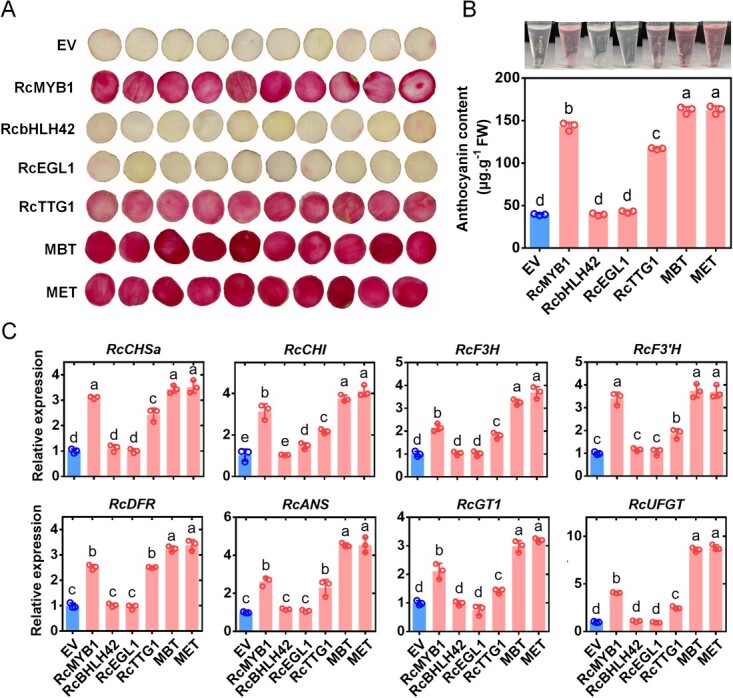
Transient overexpression of MBW complexes alone or in combination in rose petals. **A** Phenotypes of petals expressing different constructs. EV, empty vector. Other expressed rose genes encoding MYB1, bHLH42, EGL1, TTG1, and two MBW complexes (MBT and MET). **B** For (A) anthocyanin content determination. **C** Relative expression of anthocyanin-related genes after vacuuming for 5 d. Values are means ± SDs (*n* = 3). Lowercase letters, (a–d) in **B** and (a–e) in **C**, indicate significantly different values (Student’s *t* test, *P* < 0.05).

We further verified this result in the white petals of rose flowers. Transient overexpression of *RcbHLH42* and *RcEGL1* alone did not promote anthocyanin accumulation (Fig. 5A and B), Those expressing each of the MBW complexes accumulated more anthocyanins than did the line expressing *RcMYB1* alone, but there was no significant difference in anthocyanin content between the two MBW complex lines (Fig. 5A and B). In contrast to the results of transient overexpression in tobacco leaves, white petals transiently expressing *RcTTG1* alone showed a significant increase in anthocyanin accumulation (Fig. 5A and B). Further analyses revealed that transient overexpression of *RcTTG1,* but not *RcbHLH*s (*RcbHLH42* or *RcEGL1*), increased the transcript levels of ABGs (Fig. 5C). The transcript levels of ABGs were higher in the groups expressing the two MBW complexes than in those expressing *RcMYB1* and *RcTTG1* alone (Fig. 5C). We detected higher transcript levels of both EBGs and LBGs in the groups expressing the two MBW complexes than in the group expressing *RcMYB1* alone. Previous studies have shown that MBW complexes generally regulate LBGs. Thus, we hypothesized that the MBW complexes could enhance the expression of *RcMYB1* to regulate the expression of EBGs. In addition, there were no significant differences in phenotype and regulation of ABGs between the two lines expressing MBW complexes, indicating that the two complexes are functionally redundant.

The phenotypes of transgenic tobacco lines also confirmed our results. The *35S:RcbHLH42* and *35S:RcEGL1* transgenic lines had the same flower color as the wild-type (WT), with no significant accumulation of anthocyanins (Fig. S8C and D, see online supplementary material). The *35S:RcMYB1* transgenic line had deeper flower color and significant accumulation of anthocyanins (Fig. S8C and D, see online supplementary material). Unlike the transient expression results, the *35S:RcTTG1* transgenic line had deeper flower color compared with that of WT (Fig. S8C and D, see online supplementary material). There was no significant difference in anthocyanin accumulation between the two transgenic lines expressing the MBW complexes, but both of them had deeper flower color and accumulated more anthocyanins compared with the *35S:RcMYB1* and *35S:RcTTG1* transgenic lines (Fig. S8C and D, see online supplementary material).

### RcMYB1 alone or in MBW complexes activates its own promoter and those of other anthocyanin biosynthesis genes

**Figure 6:**
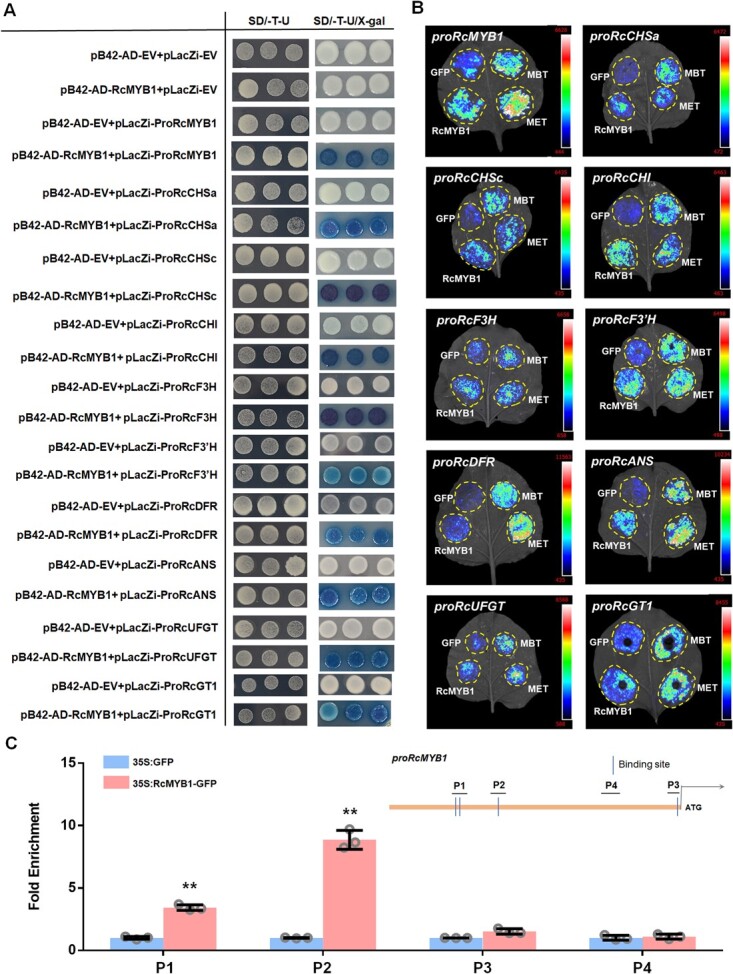
Analyses of interactions between MYB1 and promoters of various genes involved in anthocyanin biosynthesis. **A** Y1H assays showing the binding of *RcMYB1* proteins to the promoters of *RcMYB1*, *RcCHSa*, *RcCHSc*, *RcCHI*, *RcF3H*, *RcF3′H*, *RcDFR*, *RcANS*, *RcUFGT*, *RcGT1*. Yeast cells were cultured on selective medium (SD-Trp-Ura/80 mg/L X-gal). The blue precipitate represents cumulative beta-galactosidase activity resulting from LacZ reporter binding activation. Three representative colonies are shown for each binding. **B** LUC assays verified the transcriptional activation ability of *RcMYB1* and two rose MBW complexes (MBT and MET) towards the *RcMYB1* and ABGs. CCD imaging system was used to capture luminous images. Color scale indicates LUC signal intensity (red, strong; blue, weak). **C** ChIP-qPCR assay for the direct binding of RcMYB1 to *RcMYB1* promoter. Values are means ± SDs (*n* = 3). Asterisks indicate significantly different values (Student’s *t* test, ^**^P < 0.01).

To further investigate the role of RcMYB1 in regulating ABGs, we performed Y1H and dual-luciferase reporter (LUC) assays. When pLacZi-*proRcMYB1*, *proRcCHSa, proRcCHSc, proRcCHI, proRcF3H, proRcF3′H, proRcDFR, proRcANS, proRcUFGT* or *proRcGT1* fusion constructs were co-expressed with pB42AD-*RcMYB1* in yeast cells, the yeast cells turned blue when screened on the selective medium (SD/−Trp/-Ura) supplemented with X-gal. The negative control (BD) failed to turn blue on the selective medium (Fig. 6A). These results showed that RcMYB1 is able to bind not only to its own promoter but also to those of a wide range of ABGs (EBGs and LBGs).

Next, we performed LUC experiments to determine whether RcMYB1 and the two MBW complexes activate the promoters of *RcMYB1* and ABGs. The results showed that RcMYB1 can activate its own promoter, and the transcriptional activity was further enhanced by the two MBW complexes (Fig. 6B). ChIP-qPCR further confirmed the interaction between RcMYB1 and its own promoter *in vivo* (Fig. 6C). In addition, RcMYB1 also significantly activated the promoters of all of EBGs and LBGs. The two MBW complexes further enhanced the promoter activity of LBGs, but not those of EBGs (Fig. 6B). These results suggested that RcMYB1 can activate its own promoter and those of a wide range of ABGs, while the two MBW complexes enhance the promoter activity of *RcMYB1* and LBGs.

### RcMYB1 *participate in carotinoids and volatile aroma metabolism but not in PA metabolism*

Some studies have indicated that MYB-TFs can regulate the accumulation of anthocyanins and proanthocyanidins (PA), and also play an important regulatory role in a variety of secondary metabolic pathways. We found that *RcMYB1* has a high expression level in yellow flowers and fragrant white flowers (Fig. 1D and E). Furthermore, we found potential MYB binding sites in these metabolic pathways genes promoters. Therefore, we studied whether *RcMYB1* was involved in these metabolic pathways. Our results showed that the expression of *Leucidin reductase* (*LAR*) or *Anthin reductase* (*ANR*), key enzymes involved in PA synthesis [30], were not increased after overexpression of *RcMYB1* and two MBW complexes (Fig. S9A, see online supplementary material). Y1H and Luc assays also indicated that RcMYB1 is not involved in transcriptional regulation of *RcLAR* and *RcANR* (Fig. S10A and B, see online supplementary material).

**Figure 7:**
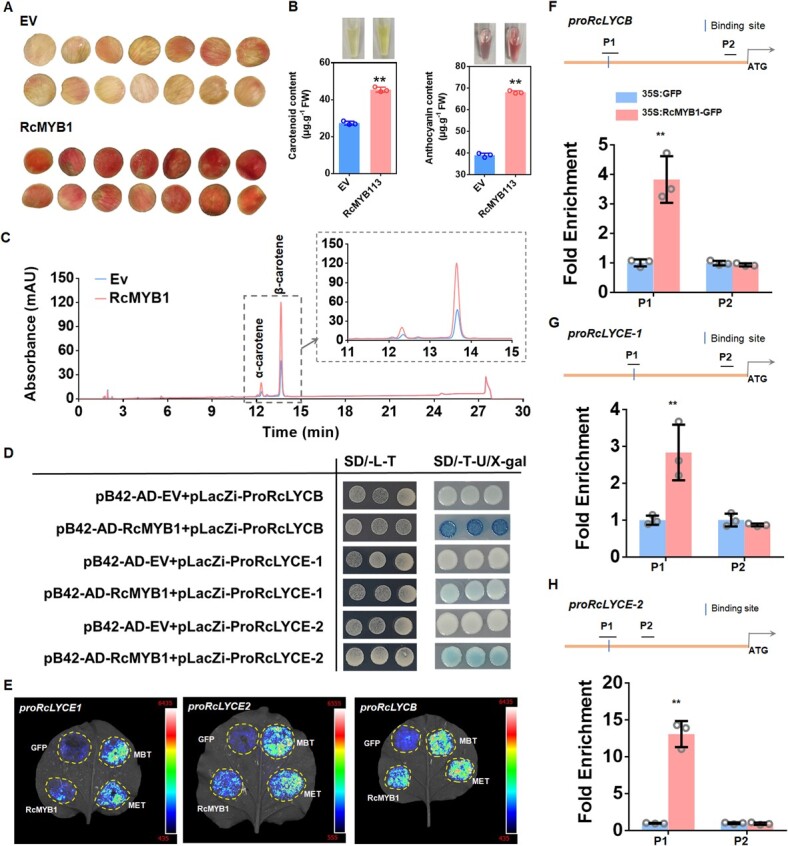
RcMYB1 regulated the biosynthesis of α-carotene and β-carotene. **A** Phenotypes of *Rosa hybrida* ‘Lady of Shalott’ petals after over-expressing RcMYB1. **B** Carotenoids and anthocyanins content determination. **C** The determination of α-carotene and β-carotene contents after overexpression *RcMYB1* by UPLC. **D** Y1H assays showing the binding of *RcMYB1* proteins to the promoters of *RcLYCB*, *RcLYCE*-*1* and *RcLYCE*-*2*. Yeast cells were grown on selective medium (SD-Trp-Ura/80 mg/L X-gal). The blue precipitate represents cumulative beta-galactosidase activity resulting from LacZ reporter binding activation. Three representative colonies are shown for each binding. **E** LUC assays verified the transcriptional activation ability of *RcMYB1* and two rose MBW complexes (MBT and MET) towards the *RcLYCB*, *RcLYCE*-*1*, and *RcLYCE*-*2*. CCD imaging system was used to capture luminous images. Color scale indicates LUC signal intensity (red, strong; blue, weak). **F** ChIP-qPCR assay for the direct binding of RcMYB1 to *RcLYCB* promoter. **G** ChIP-qPCR assay for the direct binding of RcMYB1 to *RcLYCE*-*1* promoter. **H** ChIP-qPCR assay for the direct binding of RcMYB1 to *RcLYCE*-*2* promoter. Values in B, F, G, H are means ± SDs (*n* = 3). Asterisks in B, F, G, H indicate significantly different values (Student’s *t* test, ^**^P < 0.01).

Lycopene beta cyclase (LCYB) and lycopene εcyclase (LCYE) are key branching points in the carotenoid synthesis pathway [34]. Interestingly, we found that the expression level of *RcLYCB*, *RcLYCE-1*, and *RcLYCE-2* were significantly increased after overexpression of *RcMYB1*, and further enhanced by two MBW complexes (Fig. S9B, see online supplementary material). In order to further study the relationship between RcMYB1 and carotenoid synthesis, transient overexpression was performed in *R. hybrida* ‘Lady of Shalott’, which had light orange petals. The results showed that the petals showed a deeper orange-red color after overexpression of *RcMYB1* (Fig. 7A), and the contents of carotenoids and anthocyanins in petals were significantly increased (Fig. 7B). Further, UPLC was used to determine the changes of α-carotene and β-carotene contents after overexpression *RcMYB1*. The results showed that both α-carotene and β-carotene contents were significantly increased after *RcMYB1* overexpression in *R. hybrida* ‘Lady of Shalott’ (Fig. 7C). To further verify whether RcMYB1 directly regulated the transcription of *RcLYCB*, *RcLYCE*-*1* and *RcLYCE*-*2*, Y1H, Luc, and ChIP-qPCR assays were conducted. Our results showed that RcMYB1 could directly bind and activate the promoter of *RcLYCB*, *RcLYCE*-*1*, and *RcLYCE*-*2* (Fig. 7D and E). ChIP-qPCR also showed that RcMYB1 could significantly enrich the binding sites on promoters of *RcLYCB*, *RcLYCE*-*1*, and *RcLYCE*-*2* (Fig. 7F, G, H).

**Figure 8:**
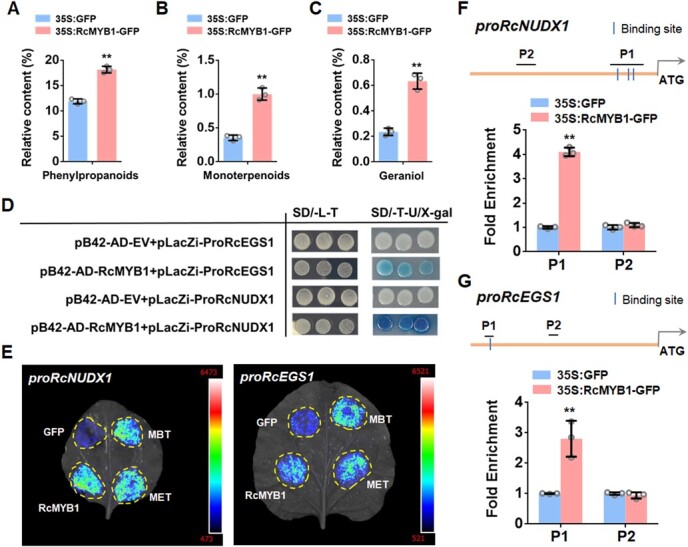
RcMYB1 regulated the geraniol and eugenol biosynthesis in rose. **A** GC-TOFMS analysis of the relative contents of phenylpropanoids after overexpression of *RcMYB1*. **B** GC-TOFMS analysis of the relative contents of monoterpenoids after overexpression of *RcMYB1*. **C** GC-TOFMS analysis of the relative contents of geraniol after overexpression of *RcMYB1*. **D** Y1H assays showing the binding of RcMYB1 proteins to the promoters of *RcNUDX1* and *RcEGS1*. Yeast cells were grown on selective medium (SD-Trp-Ura/80 mg/L X-gal). The blue precipitate represents cumulative beta-galactosidase activity resulting from LacZ reporter binding activation. Three representative colonies are shown for each binding. **E** LUC assays verified the transcriptional activation ability of *RcMYB1* and two rose MBW complexes (MBT and MET) towards the *RcNUDX1* and *RcEGS1*. CCD imaging system was used to capture luminous images. Color scale indicates LUC signal intensity (red, strong; blue, weak). **F** ChIP-qPCR assay for the direct binding of RcMYB1 to *RcNUDX1* promoter. **G** ChIP-qPCR assay for the direct binding of RcMYB1 to *RcEGS1* promoter. Values in **A**, **B**, **C**, **F**, and **G** are means ± SDs (*n* = 3). Asterisks in **A**, **B**, **C**, **F**, and **G** indicate significantly different values (Student’s *t* test, ^**^P < 0.01).

In modern roses, *RhNUDX1* is involved in the formation of geraniol, the main rose fragrance compound [35]. *RcEGS1* is responsible for eugenol biosynthesis in rose [36]. The expression of *RcEGS1* and *RcNUDX1* was also increased after overexpression of *RcMYB1* and two MBW complexes (Fig. S9C, see online supplementary material). In order to further investigate whether RcMYB1 is involved in the metabolism of aromatic compounds, GC-TOFMS analysis was performed on the transient overexpression *RcMYB1* in *R. hybrida* ‘Gabriel’. After overexpression of *RcMYB1*, the relative contents of the main aromatic substances phenylpropanoids and monoterpenoids were significantly increased (Fig. 8A and B). Geraniol belongs to monoterpenoids, which is synthesized by *RcNUDX1*. In our results, we also found a significant increase in the relative content of geraniol (Fig. 8C). However, eugenol was not found in our determination results. Eugenol is in phenylpropanoids, which commonly present in rose stamens but not in rose petals [36]. Further, Y1H, Luc, and ChIP-qPCR assays were conducted to determine whether RcMYB1 directly regulated *RcNUDX1* and *RcEGS1*. Our results showed that RcMYB1 could directly bind and activate the promoter activity of *RcNUDX1* and *RcEGS1* (Fig. 8D, E, F, G).

## Discussion

### RcMYB1 plays an important role in regulating anthocyanin accumulation in rose

R2R3-MYBs are the most important in anthocyanin synthesis, and many R2R3-MYBs that positively regulate anthocyanin biosynthesis have been identified in various plant species. In Arabidopsis, *PAP1* makes seedlings produce anthocyanins, but *MYB113*/*114* and *PAP2* are partially redundant in anthocyanin biosynthesis [25]. Anthocyanin biosynthesis is activated by *SlAN2-like* tomatoes (*Solanum lycopersicum*) [37]. In addition, *PhAN2* in petunia [38]; *MdMYB1/10/110a*, and in apple [12, 13, 39, 40]; *PpMYB10*.*1*/*10*.*2*/ /*108* in peach [41, 42]; *SmMYB1* in eggplant [43]; *AaMYB2* in *Anthurium andraeanum* [44]; *VvMYBA1* in common grape (*Vitis vinifera*) [45]; *FhPAP1* in *Freesia hybrida* [46]; and *PcMYB10* in Chinese white pear (*Pyrus bretschneideri*) [47] have also shown a positive role in regulating anthocyanin biosynthesis. In our study, we identified the RcMYB1 (R2R3 MYB) as a key activator that promotes anthocyanin biosynthesis in rose. Overexpression of *RcMYB1* strongly promoted the accumulation of anthocyanins in white petals, leaves, and petioles (Figs 2 and 3).

MYB-TFs can directly bind to the promoters of ABGs to activate their transcription. PpMYB108 regulates peach anthocyanin biosynthesis by binding to *PpDFR* promoter [41]. AaMYB2 promotes anthocyanin accumulation in *A. andraeanum* by regulating the expression of *AaCHS*, *AaF3H*, and *AaANS* [44]. In golden kiwifruit (*Actinidia chinensis*), AcMYBF110 binds to the *AcCHS*, *AcF3′H*, *AcANS*, *AcUFGT3a*/*T6b*, but not to the promoters of *AcCHI*, *AcF3H*, or *AcDFR* [48]. However, in our study, we found that RcMYB1 broadly binds and activates the promoters of EBGs (*RcCHSa*, *RcCHSc*, *RcCHI*, *RcF3H*, and *RcF3’H*) and LBGs (*RcDFR*, *RcANS*, *RcUFGT*, and *RcGT1*) in the anthocyanin pathway (Fig. 6).

In our study, we found that *RcMYB1* is involved in an autoregulatory feedback loop to regulate the accumulation of anthocyanins (Fig. 6). A similar autoregulatory feedback loop has also been reported in apple *MdMYB10* and monkeyflower (*Mimulus lewisii*) *PELAN* (*Petal Lobe Anthocyanin*) [13, 49]. Some inhibitory R2R3-MYBs have also been shown to be under autoregulatory control. *Arabidopsis MYB4* can inhibit its own transcription by binding to its own promoter as part of a negative autoregulatory loop [50]. Similar to rice (*Oryza sativa*) *OsMYB4* [51] and petunia *MYB27* [19].

### RcbHLH42, RcEGL1, and RcTTG1 are involved in the regulation of anthocyanin accumulation by interacting with RcMYB1

In this study, we constructed a phylogenetic tree containing all rose bHLH family proteins as well as documented bHLH proteins (MYC1/EGL3/GL3/TT8) related to anthocyanin biosynthesis in *Arabidopsis*. We found that only RcbHLH42 and RcEGL1 were closely related to these four bHLH proteins in *Arabidopsis* (Fig. S4, see online supplementary material). bHLHs typically play a role in the formation of MBW complexes and enhance MYB activity but do not promote anthocyanin biosynthesis alone [52]. Consistent with the finding that FhGL3L and FhTT8L of *F. hybrida* cannot regulate anthocyanin biosynthesis independently of endogenous MYB proteins [53], we did not observe anthocyanin accumulation after overexpressing *RcbHLH42* and *RcEGL1* alone in tobacco leaves and rose petals (Fig. 5A; Fig. S8A, see online supplementary material). However, in *Arabidopsis*, the mutation of *TT8* reduced anthocyanin and made seed coat colorless [54]. As *RcMYB1* can independently activate EBGs and LBGs and can strongly promote the accumulation of anthocyanin in tobacco leaves and white rose petals, we think that the accumulation process of anthocyanin in rose petals mediated by *RcMYB1* may not depend on *RcbHLH42* and *RcEGL1*. In addition, after forming the MBW complex with RcMYB1 and RcTTG1, the transcriptional activity of *RcMYB1* was enhanced by two MBW complexes (MBT, MET), so *RcbHLH42* and *RcEGL1* may play a role as enhancers—mediated by *RcMYB1*—in anthocyanin accumulation in rose petals.

A few WDR are involved in anthocyanin synthesis; on example is the WD40 protein TTG1, which has been isolated from a number of plant species [55]. In *Arabidopsis*, lack of seed coat pigment and decrease of anthocyanin accumulation in *ttg1* mutant [56]. In rice, the anthocyanin content in the *osttg1* mutant was significantly reduced in various organs [57]. Overexpression of *CsWD40* in tobacco resulted in significant increase of anthocyanin content in petals [4]. In our study, anthocyanins accumulated significantly after *RcTTG1* was overexpressed in white rose petals (Fig. 5A), which showed that *RcTTG1* played an important role in the accumulation of anthocyanins in rose.

In the MBW complex, bHLH proteins usually interact with both MYB and WDR as linkers, while WDR may not interact with MYB. In apple, MdTTG1 interacts with bHLH but not with MYB proteins [58]. In *Paeonia qiui*, PqMYB113 interacts with PqbHLH1 but not with PqWD40 [52]. In tomato, SlAN11 (WDR) interacts with bHLH but not with the MYB protein in the MBW complex [59]. However, in our study, we found that RcTTG1 can interact not only with RcbHLHs but also with the RcMYB1 protein (Fig. 5). The WD40 protein interaction with MYB protein has also been reported in recent studies. For example, in woodland strawberry, FcTTG1 can interact with FcMYB114, FcMYB123, and FcbHLH42 [60]; in eggplant SmMYB86 interacts with SmTTG1 [61]; in *Medicago truncatula* MtWD40–1 interacts with MtPAR or MtLAP1 [62]; and in kiwifruit AcWDR1 interacts with AcMYBF110 [48].

The MBW complex is the key factor in the regulation of LBGs [28]. In Arabidopsis, EBGs are activated by coactivator-independent and functionally redundant R2R3-MYBs (MYB11/12/111), whereas activation of LBGs requires a MBW complex [25]. Similar to our results, we found that two MBW complexes significantly enhanced the activity of LBGs but had only a slight effect on EBGs activity (Fig. 6B). However, some studies have revealed that all ABGs are regulated by MBW complexes, such as in maize (*Zea mays*) [63], *F. hybrida* [46], rice (*O. sativa*) [64], and sweet potato [65]. These results suggest that MBW complexes may have different regulatory mechanisms for anthocyanin accumulation.

### RcMYB1-RcbHLH42-RcTTG1 (MBT) and RcMYB1-RcEGL1-RcTTG1 (MET) are functionally redundant in anthocyanin accumulation in rose

The MBW complex has been shown to have functional redundancy for anthocyanidin biosynthesis in many plants [55]. Examples include AtTT8, AtGL3, and AtEGL3 in *Arabidopsis* [25], MdbHLH3/33 in apple [13], LcbHLH1/3 in *Litchi chinensis* [66], and FhGL3L and FhTT8L in *F. hybrida* [53]. In this study, we identified two MBW complexes (MBT, MET) that can promote anthocyanin accumulation. Based on ABGs expression level and anthocyanin content (Fig. 5A), we found that these two complexes may have functional redundancy in the regulation of anthocyanidin biosynthesis.

In addition, we found that the transcript levels of *RcbHLH42* and *RcEGL1* were not associated with anthocyanin accumulation during rose flower development, consistent with previous observations for *LcbHLH1*/*2*/*3* in *L. chinensis* [66]; for *MdbHLH33*/*3* in apple [13]; for *VvMYC1* in grape [45]; and for *FhGL3L* in *F. hybrida* [53]. *RcEGL1* was only highly expressed at the S1 stage and gradually decreased with the development of flower buds, while *RcbHLH42* was highly expressed at the S3 and S4 stages (Fig. S5C, see online supplementary material), so we speculated that the MET complex might play a role in anthocyanin synthesis at an earlier stage than the MBT complex. In addition, we also found that the expression of *RcEGL1* increased after MBT complex overexpression, and the expression of *RcbHLH42* also increased after MET overexpression (Fig. S11, see online supplementary material). Therefore, we speculated that there might be cooperation in the regulatory mechanism in these two MBW complexes.

### RcMYB1 is involved in other secondary metabolic pathways

So far, the roles of R2R3-MYBs in plants are mainly (∼70%) in regulating plant metabolic processes, including the benzenoid, phenylpropanoid, terpenoid, and glucosinolate (GSL) pathways [67]. In this study, we found that RcMYB1 is involved in multiple secondary metabolic pathways. Previous studies have found that MYB regulates anthocyanin and proanthocyanidin biosynthesis. Such as *MtMYB5*/*14* in *M. truncatula* [68], *MdMYB9*/*11* in apple [69], *RrMYB5*/*10* in *R.rugosa* [33]. However, in this study, we found that *RcMYB1* did not regulate the transcription of *RcLAR* and *RcANR* (Figs S8A and S9, see online supplementary material).

Interestingly, we found that *RcMYB1* regulates the biosynthesis of carotenoids and aromatic volatiles. Carotenoids are closely related to the coloration of yellow, orange red or red flowers [70]. Lycopene beta cyclase (LCYB) and lycopene εcyclase (LCYE) are key branching points in the carotenoid synthesis pathway [34]. In this study, we transient overexpressed *RcMYB1* in *R. hybrida* ‘Lady of Shalott’, and found that petals changed from light orange to orange-red (Fig. 7A), further we confirmed that RcMYB1 can directly regulate the transcription of *RcLYCB*, *RcLYCE*-*1*, and *RcLYCE*-*2*, thereby mediating the accumulation of α-carotene and β-carotene (Fig. 7). Many studies have reported that R2R3 MYB TFs can directly regulate carotenoid biosynthesis. For instance, in *M. truncatula*, WHITE PETAL1 (WP1) directly regulates the expression of *MtLYCe* and *MtLYCb* [71]. AdMYB7 can activate the promoter of *AdLCYB* in *A. chinensis* [34]. Similarly, in *Camellia sinensis* [72], in *Rhyncholaeliocattleya* [73], and in *M. lewisii* [74] have also been reported.

In addition, many R2R3 MYB-TFs have also been found to contribute to volatile aroma metabolism and control the emission of volatile substances [75]. A regulatory network of three R2R3 MYB-TFs, ODORANT1 (ODO1), EMISSION OF BENZENOIDS I (EOBI), and EOBII, can directly activate the transcription of many genes in the shikimic acid pathway and its downstream floral synthesis pathway [76, 77]. Expression of *AtPAP1* not only promotes the synthesis of phenylpropanoid and terpenoid odour compounds but also promotes the accumulation of surfactants in *R. hybrida*, tobacco, and *petunia* [78]. Aroma components such as aldehydes, phenylpropanoids, and terpenes increased significantly in *SlMYB75*-OE tomato fruits [79]. In *A. thaliana*, MYB3 binds to the promoter of the gene *cinnamate 4*-*hydroxylase* (*C4H*) to negatively regulate gene expression and inhibit phenylpropanoid synthesis [80]. PhMYB4 has also been reported to inhibit the expression of *PhC4H* in *P. hybrida* [81]. In rose, a 1R-MYB TF, *RhMYB1* may be a putative gene involved in the biosynthesis of rose scent [82]. Here, we provide evidence that a R2R3 MYB TF, RcMYB1, is involved in the biosynthesis of eugenol and geraniol in rose (Fig. 8). Phenylpropanoids and monoterpenoids are the main floral compounds of rose. After overexpression of *RcMYB1*, we found that the content of phenylpropanoids and monoterpenoids showed significant increases, and the content of geraniol, catalyzed by RcNUDX1, also significantly increased. Our determination results did not detect eugenol, catalyzed by RcEGS1, possibly because eugenol is normally present in the stamens of rose [36]. Interestingly, the contents of many phenylpropanoids and monoterpenoids were significantly increased after overexpression of *RcMYB1*, suggesting that RcMYB1 may also be widely regulated in the metabolism of aromatic compounds.

### Conclusion

In conclusion, we revealed that R2R3-MYB *RcMYB1* plays a central role in anthocyanin biosynthesis and also contributes to the metabolism of carotenoids and aromatic volatiles. Two bHLH proteins (*RcbHLH42* and *RcEGL1*) can bind *RcMYB1* and *RcTTG1* to form the MBW complex, mainly regulating LBGs to regulate anthocyanin biosynthesis.

## Materials and methods

### Plant materials

*Rosa chinensis* and *R. hybrida* roses cultivars were planted at the Germplasm Resource Center of Shanghai Normal University, Shanghai, China. Petals were sampled at seven developmental stages (S1–S7) from *R. chinensis* ‘Old Blush’. Petals were also sampled on the day of flowering from the following: three blue-purple rose cultivars (*R. hybrida* ‘Lavender Bouquet’, *R. hybrida* ‘Libellula’, and *R. hybrida* ‘Kong Meng’); six red rose cultivars (*R. hybrida* ‘Muriel Robin’, *R. hybrida* ‘Angela’, *R. hybrida* ‘Yan Li’, *R. hybrida* ‘Ren Yue’, *R. hybrida* ‘Cherry Bonica’, and *R. hybrida* ‘Black Magic’); one green rose cultivar (*R. hybrida* ‘Duo Lei’); one yellow rose cultivar (*R. hybrida* ‘Yellow Leisure Liness’); and one white rose cultivar (*R. hybrida* ‘Gabriel’). The petals were divided into three replicates, frozen in liquid nitrogen and stored at −80°C.

### *Cloning of the* RcMYB1*,* RcbHLH*s* (RcbHLH42 *and* RcEGL1), *and* RcTTG1 *genes and the promoters of* RcMYB1 *and anthocyanin biosynthesis genes*

The full-length sequences of *RcMYB1*, *RcbHLH42*, *RcEGL1*, and *RcTTG1* were cloned from the cDNA of *R. chinensis* ‘Old Blush’. The promoter sequences of *RcMYB1* (2230 bp), *RcCHSa* (987 bp), *RcCHSc* (578 bp), *RcCHI* (826 bp), *RcF3H* (615 bp), *RcF3′H* (557 bp), *RcDFR* (522 bp), *RcANS* (2128 bp), *RcGT1* (2053 bp), and *RcUFGT* (1986 bp) were also cloned from the DNA of *R. chinensis* ‘Old Blush’. The primers used are shown in Table S1 (see online supplementary material).

### Sequence alignment and phylogenetic analysis

The full-length amino acid sequences of RcMYB1, RcbHLH42, RcEGL1, and RcTTG1 in rose were aligned using CLUSTAL OMEGA. The amino acid sequences of MYB, bHLHs, and WD40 previously identified were from NCBI GenBank. RcMYB1, RcbHLH42, RcEGL1, and RcTTG1 were functionally classified by phylogenetic tree analysis. MEGA11 (https://megasoftware.net/) and iTOL (https://itol.embl.de/) were used to build a maximum likelihood tree to analyse the phylogenetic relationship between protein.

### RT–qPCR

Total RNA was extracted using the SteadyPure Plant RNA Extraction Kit (Accurate Biology, Hunan, ChangSha, China). qPCR was performed using Hifair® III 1st Strand cDNA Synthesis SuperMix (Yeasen, Shanghai, China) PerfectStart® Green qPCR SuperMix (Transgen Biotech, Beijing, China) was used for RT-qPCR. The 2^−ΔΔCt^ method was used, with RcActin as the internal parameter. The primers of RT-qPCR are shown in Table S2 (see online supplementary material).

### Subcellular localization

The ORFs of *RcMYB1, RcbHLH42, RcEGL1*, and *RcTTG1* (without stop codons) were cloned into pCAMBIA 2300 and transformed into *Agrobacterium tumefaciens*. The solution (10 mM MgCl_2_, 10 mM methylester sulfonate, and 200 μM acetosyringone, pH 5.7) was used to prepare an OD_600_ = 1.0 infiltration buffer to infiltrate 6-week-old *N. benthamiana* leaves. After 3 d, with 150 mu g/mL DAPI (4',6-diamidino-2-phenylindole) staining. Fluorescence images of GFP and DAPI were observed under 488 nm and 340 nm excitation light (Olympus FV3000 confocal scanning microscope, Olympus, Tokyo, Japan), respectively.

### Transient overexpression in R. hybrida ‘Gabriel’, R. hybrida ‘Lady of Shalott’, and N. tabacum

For the transient assay in *R. hybrida* ‘Gabriel’ and *R. hybrida* ‘Lady of Shalott’ petal discs, *A. tumefaciens*-mediated vacuum infiltration was used. OD_600_ = 1.0, as described above. The petals were immersed in the infection solution, then treated in a vacuum of −80 kPa for 5 minutes, twice, placed in a filter paper petri dish containing 1% sucrose at 18°C for 6 d. Each treatment contained three experimental replicates, and approximately 100 petal discs were used from five flowers.

For the transient assay in *N. tabacum* leaves, the above different combinations of strains infected leaves, 7 days sampling.

### *Transformation of R. hybrida* ‘Novalis’ *and N. tabacum*

Transformation of *R. hybrida* ‘Novalis’ was performed via *A. tumefaciens*-mediated transformation using leaf-derived embryogenic callus [83]. Briefly, sterile young leaves were used to induce callus on the callus induction medium (CIM) and further induce somatic embryos on the embryos induction medium (EIM). *A. tumefaciens* cells containing pCAMBIA 2300-*RcMYB1* were collected and suspended at OD_600_ = 0.5. Then they were washed three times in an infiltrating solution at −80 kPa for 15 min and cocultured on a co-culture medium (CM) at 22°C dark for 4 days. Somatic embryos were cultured on screening medium supplemented with kanamycin and timentin, and positive transgenic plants were identified by genomic PCR, RT-q PCR, and Western blotting.

*A. tumefaciens* cells containing *RcMYB1*, *RcbHLH42*, *RcEGL1*, or *RcTTG1* were transformed into *N. tabacum* leaves using the leaf disc method [84]. Transgenic plants were selected for each construct in a medium containing kanamycin and timentin. Then transfer to the greenhouse until flowering. Positive transgenic (genomic PCR and RT-q PCR) T1 progeny tobacco plants were identified.

### YIH

The CDS of RcMYB1 was inserted into the pB42AD vector, and the promoter and anthocyanin synthesis genes of *RcMYB1* were inserted into the pLacZi vector. Strain Y1HGold (Clontech, Mountain View, CA, USA) was used. PEG/LiAc method was used for conversion. The transformed yeast cells were inoculated into (SD/−Trp/-Ura) medium with or without X-Gal. Use the primers in Table S3 (see online supplementary material).

### Y2H

Insert CDS of RcMYB1, RcbHLH42, RcEGL1, and RcTTG1 into the pGADT7 or pGBKT7 carrier. The plasmid was transformed into Y2HGold (Clontech, Mountain View, CA, USA) cells and then grown on SD/−Leu/−Trp medium. The potential physical interactions between proteins were further tested on SD/−Trp/−Leu/-His/−Ade medium. The primers used are shown in Table S3 (see online supplementary material).

### BiFCassay

The coding sequences of *RcMYB1* and *RcTTG1* were constructed into PXY106, and the coding sequences of *RcbHLH42*, *RcEGL1*, and *RcTTG1* were constructed into PXY104. The primers are in Table S3 (see online supplementary material). As mentioned above, infect the *N. benthamiana* leaves. After 3 d infection, the cells were stained with 150 μg/mL DAPI. Yellow fluorescent protein (YFP) fluorescence and DAPI fluorescence (Olympus FV3000 confocal scanning microscope) were observed at excitation wavelengths of 505 nm and 340 nm, respectively.

### Co-IP assay

*RcMYB1* and *RcTTG1* were cloned into PEG104 with Flag tag by Gateway™ LR Clonase™ II Enzyme mix. *RcMYB1*, *RcbHLH42*, and *RcEGL1* were cloned into PEG104 with Myc tag. Primers are in Table S3 (see online supplementary material). In Co-IP, *A. tumefaciens* cells containing different structures were collected, as described above, OD_600_ = 1.0, and then infiltrated *N. benthamiana* leaves. After 3 days, *N. benthamiana* leaves were ground at a low temperature to extract protein, then incubated at 4°C for 4 h in the presence of anti-Flag or anti-Myc antibody conjugated beads. They were rinsed four times in the wash buffer. The protein was isolated in 8% SDS-PAGE gel and analysed by Western blot.

### Anthocyanin extraction and quantification

The anthocyanin measurements followed the method described by Khazaei *et al* [85]: 2 mL extract [1% (v/v) HCl/methanol] plus a sample (FW, 0.2 g) at 4°C for 24 h under light protection, at 4°C, 12000 rpm for 1 min. Absorbance was determined by spectrophotometer (Techcomp UV-2600, Beijing, China) under A530 and A657. The calculation formula of anthocyanin relative content is as follows: ((A530 – A657) × dilution factor/mg FW tissue) × 1000.

For the determination of anthocyanins, liquid chromatography–mass spectrometry (LCMS) assays were performed on a Shimadzu LCMS-2020 (Shimadzu, Kyoto, Japan). Chromatographic separation was performed on an Agilent Zorbax SB-C18 column (4.6*250 mm, 3 μm). Eluent A (0.1% formic acid solution): Eluent B (0.1% formic acid acetonitrile solution) = 95:5. The flow rate was 1.2 mL/min, the column temperature was 30°C for 3 min, and the detection wavelength was 254 nm. Electrospray ionization (ESI) voltage was set at 0.9 KV, capillary temperature at 350°C, jacket gas pressure at 45 psi, and auxiliary gas at 10 psi. According to the retention time, the anthocyanin composition was determined, and then the anthocyanin equivalent was quantified.

### Carotenoid extraction and quantification

A 0.5 g petal sample was crushed and 2 mL anhydrous ethanol (containing 0.1%BHT) was added, then a water bath at 80°C for 5 min, adding 100 μL KOH solution (80%w/v). In a water bath at 80°C for 15 min, 1 mL purified water and 1 mL n-hexane were added, after vortex mixing, centrifuge at 3000 rpm for 5 min, transfer the supernatant, add 1 mL n-hexane into the residue again for extraction, combine the two supernatants after centrifugation, and then steam dry at 30°C under mild nitrogen. Redissolve with 0.2 mL methanol solution for detecting. The sample extracts were analysed at 450 nm using an UPLC system with DAD detector (UPLC, U3000; Thermo, Karlsruhe, Germany). The analytical conditions were as follows, UPLC: column, YMC Carotenoid S-3 μm (150*4.6 mm), column temperature, 40°C; flow rate, 1.0 mL/min; injection volume, 2 μL; solvent system, MeOH: (MeOH: MTBE: H_2_O = 20: 75: 5); gradient program, 100:0 V/V at 0 min, 39:61 V/V at 15 min, 0:100 V/V at 25 min, 100:0 V/V at 25.1 min, 100:0 V/V at 30 min.

### Identification of the volatile compounds

Volatiles were collected from petal discs by solid-phase micro-extraction (SPME) methods. Incubate 10 min at 50°C, extract 30 min, and deadsorb 5 min in GC injection mouth. After extraction, the compounds were analyzed with GC-TOFMS (Pegasus BT, Leco, St. Joseph, MI, USA). Conditions of GC-TOFMS: the temperature of DB-WAX (30 m × 250 μm × 0.25 μm) is 250°C, the interface temperature is 290°C and the ion source temperature is 230°C. The initial temperature was 40°C for 3 min, then increased to 70°C at 3°C/min, increased to 180°C at 5°C/min, and finally increased to 240°C at 10°C/min for 7 min.

### Dual-luciferase reporter assay

The LUC analysis was performed with minor modifications according to the method described by He *et al.* [87]. CDS of RcMYB1, RcbHLH42, RcEGL1, and RcTTG1 were inserted into pCAMBIA 2300 vector to form effectors. The promoters of RcMYB1 and ABGs are inserted into pGreen II-0800 to form the reporter vector. The primers used are in Table S3 (see online supplementary material). The plasmid was introduced into *A. tumefaciens* strain (containing the pSoup helper plasmid). Different *A. tumefaciens* cells composed of effector and reporter are infected into the *N.benthamiana* leaves. Three day, 20 mg/mL D-lucifin (Goldbio, St Louis, MO, USA) was sprayed on the leaves and images were collected using a cooled low-light CCD imager (Tanon-4200, Shanghai, China).

### ChIP-qPCR

ChIP-qPCR assays were performed as described by Bowler *et al.* [86]. The 5 g leaf tissues from *RcMYB1*-GFP-OE1 and *GFP*-OE plants were treated with 1% (v/v) formaldehyde to cross-link the protein-DNA complexes. The protein-DNA complexes were immunoprecipitated with an anti-GFP antibody (Cat. no. ab290; Abcam, Cambridge, UK). The immunoprecipitated DNA was purified and analysed using RT-qPCR. Use primers in Table S3 (see online supplementary material).

### Accession numbers

Gene accession numbers used in this study: *RcMYB1* (*LOC112193894*), *RcbHLH42* (*LOC112170399*), *RcEGL1* (*LOC112182591*), *RcTTG1* (*LOC112184646*), *RcCHSa* (*LOC112175474*), *RcCHSc* (*LOC112200102*), *RcCHI* (*LOC112182551*), *RcF3H* (*LOC112186620*), *RcF3′H* (*LOC112178545*), *RcDFR* (*LOC112173668*), *RcANS* (*LOC112179310*), *RcGT1* (*LOC112184328*), *RcUFGT* (*LOC112198073*), *RcLAR* (*LOC112199978*), *RcANR* (LOC112202296), *RcLYCB* (*LOC112164457*), *RcLYCE-1* (*LOC112188431*), *RcLYCE-2* (*LOC112189564*), *RcEGS1* (*LOC112201825*) and *RcNUDX1* (*LOC112189710*).

## Acknowledgments

We acknowledge Wenqiu Wang (College of Agriculture and Biotechnology, Zhejiang University, China) for the technical assistance. We thank Jennifer Smith, PhD, from Liwen Bianji (Edanz) (www.liwenbianji.cn/) for editing the English text of a draft of this manuscript.

This work was supported by Shanghai Special Project of Capacity Construction for Local Colleges and Universities, No.20070502500; Shanghai Science and Technology Agriculture Program, No.2022-02-08-00-12-F01146; Science and Technology Commission of Shanghai Municipality, No.18DZ2260500; and Shanghai Plant Germplasm Resources Engineering Research Center, 17DZ2252700.

## Author contributions

F.M. designed the research and is the author responsible for distribution of materials integral to the findings presented in this article in accordance with the policy described in the Instructions for Authors. G.H., R.Z., S.J., and H.W. conducted the experiments. G.H. and R.Z. analysed the data and wrote the manuscript. All authors read and approved the manuscript.

## Data availability

All relevant data in this study are provided in the article and its supplementary file.

## Conflict of interest

The authors declare that they have no conflict of interest.

## Supplementary data

Supplementary data is available at *Horticulture Research* online.

## Supplementary Material

Web_Material_uhad080
