# Supplementary material for: The MYB transcription factor RcMYB1 plays a central role in rose anthocyanin biosynthesis
Source: Hortic Res. 2023 Apr 21;10(6):uhad080. doi: 10.1093/hr/uhad080 (PMC10261888; doi:10.1093/hr/uhad080)
Supplement: Web_Material_uhad080 [file web_material_uhad080.zip › Revised Supplemental Figures.docx]

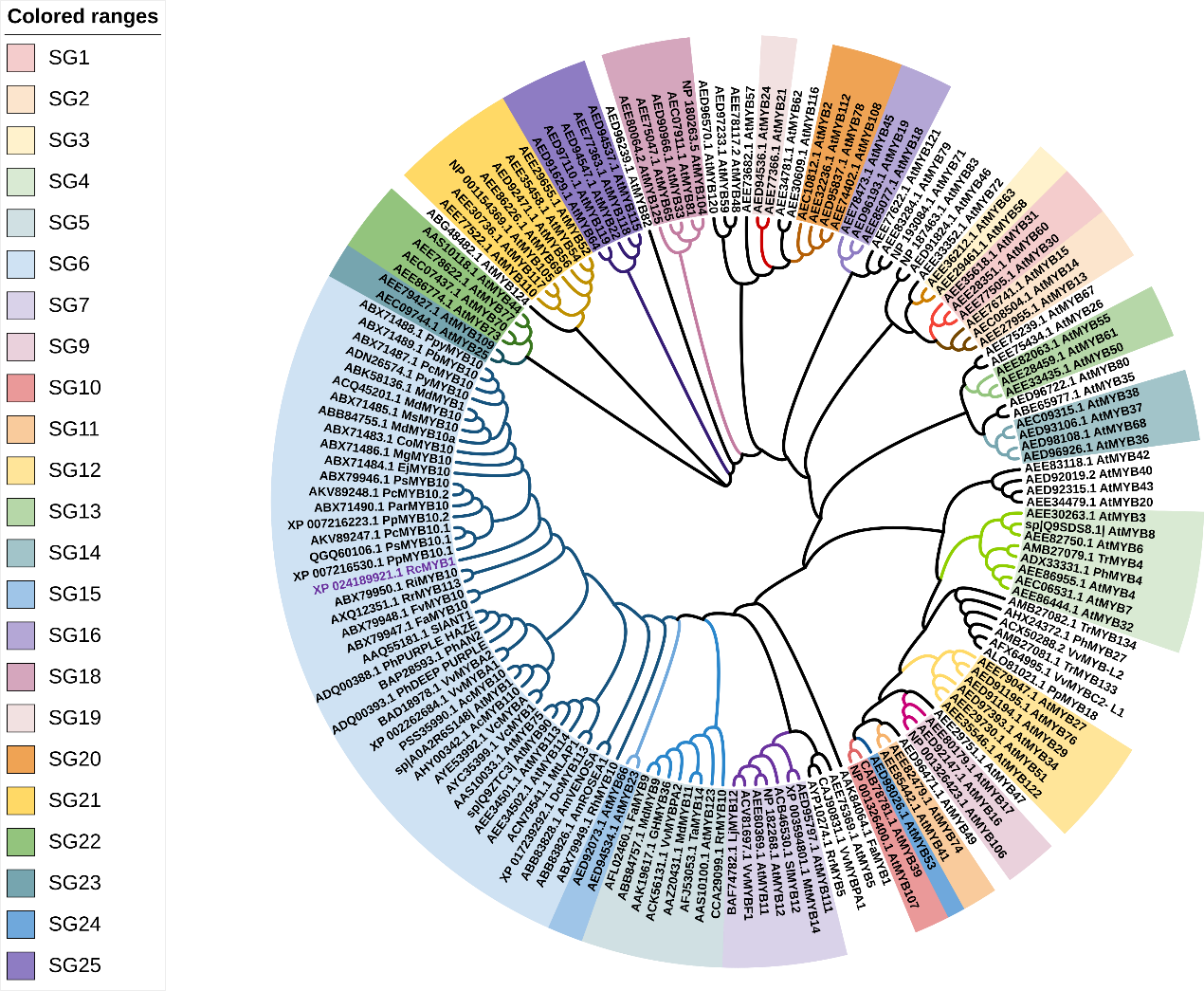


**Supplemental Figure S1.** Phylogenetic tree constructed using sequences of members of the MYB transcription factor family in *Arabidopsis* and some Rosaceae species.


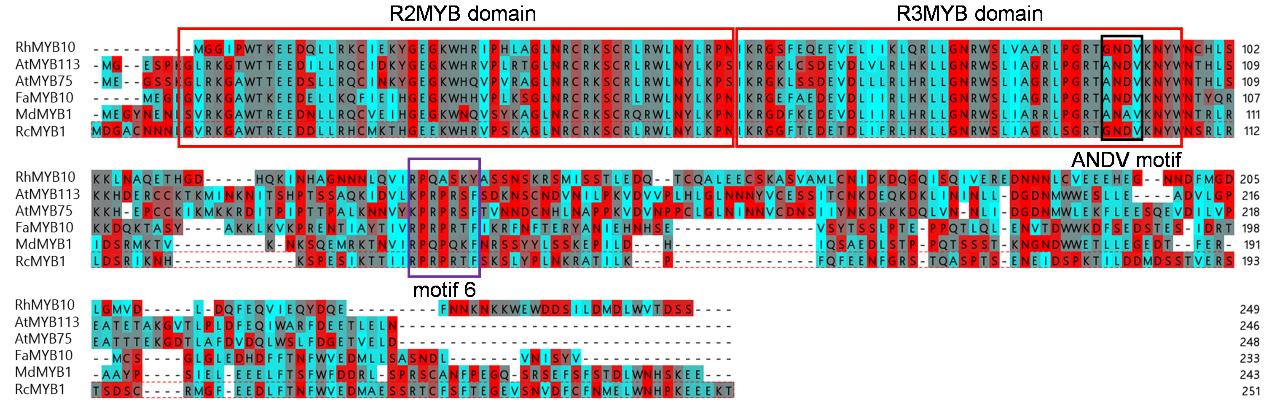


**Supplemental Figure S2.** Amino acid sequence alignment of RcMYB1 and other MYB transcription factors. The red box represents the R2 domain and the R3 domain. The black box represents the ANDV motif. The purple box represents the motif 6. *Rc*: *Rosa chinensis* (RcMYB1, XP_024189921.1), *Rh*: *Rosa hybrida* (RhMYB10, ABX79949.1), *Md*: *M*. *domestica* (MdMYB1, ABK58136.1), *Fa*: *Fragaria* × *ananassa* (FaMYB10, ABX79947.1), *At*: *Arabidopsis thaliana* (AtMYB113, AEE34501.1; AtMYB75, AAS10033.1).


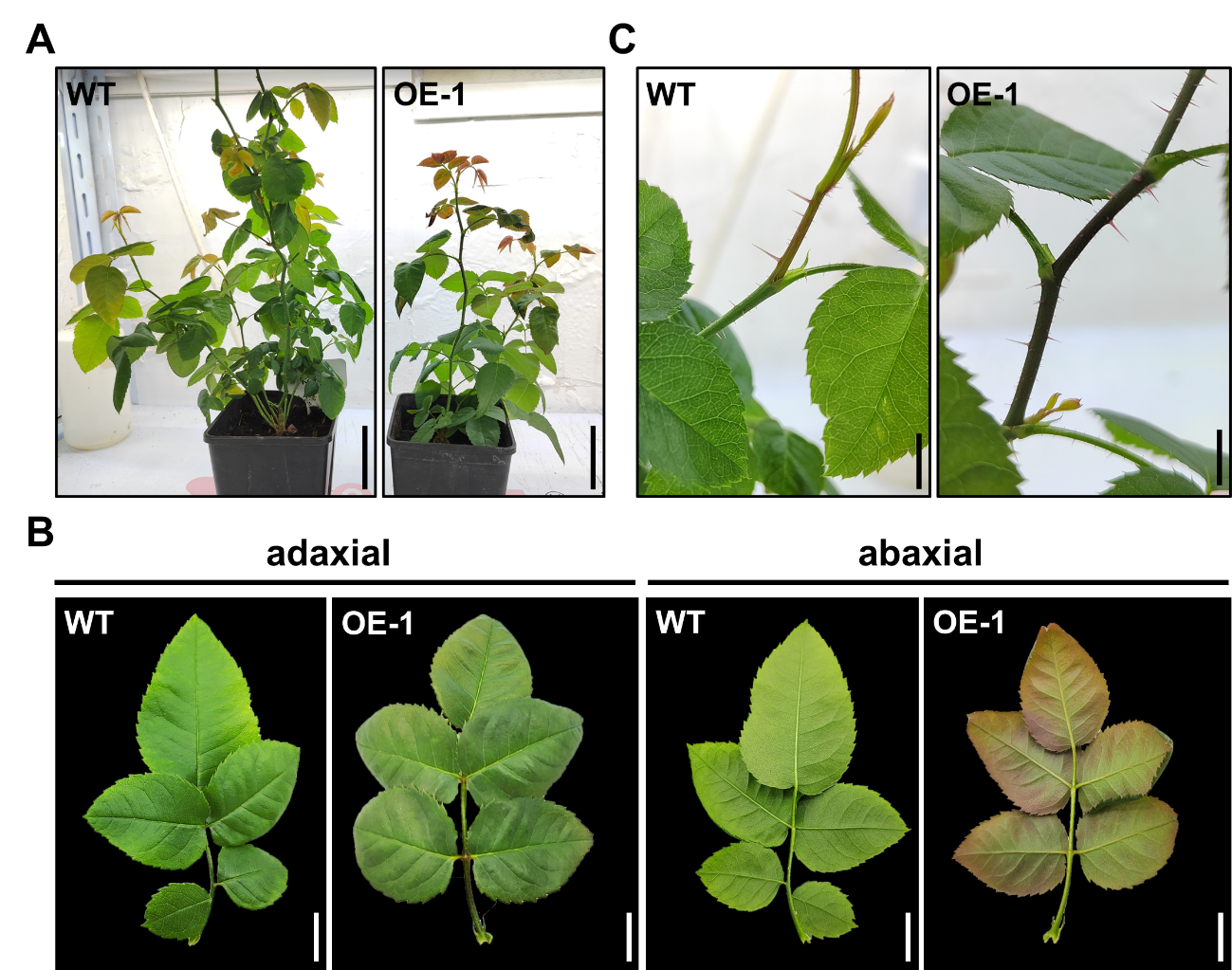


**Supplemental Figure S3.** Phenotypes of wild type (WT) and *RcMYB1*-overexpressing line. **A:** Phenotypes of wild type (WT) and OE-1 line. The scale bar is 5 cm. **B:** Phenotypes of leaf adaxial and abaxial in WT and OE-1 line. The scale bar is 1 cm. **C:** WT and OE lines stem phenotypes. The scale bar is 1 cm.


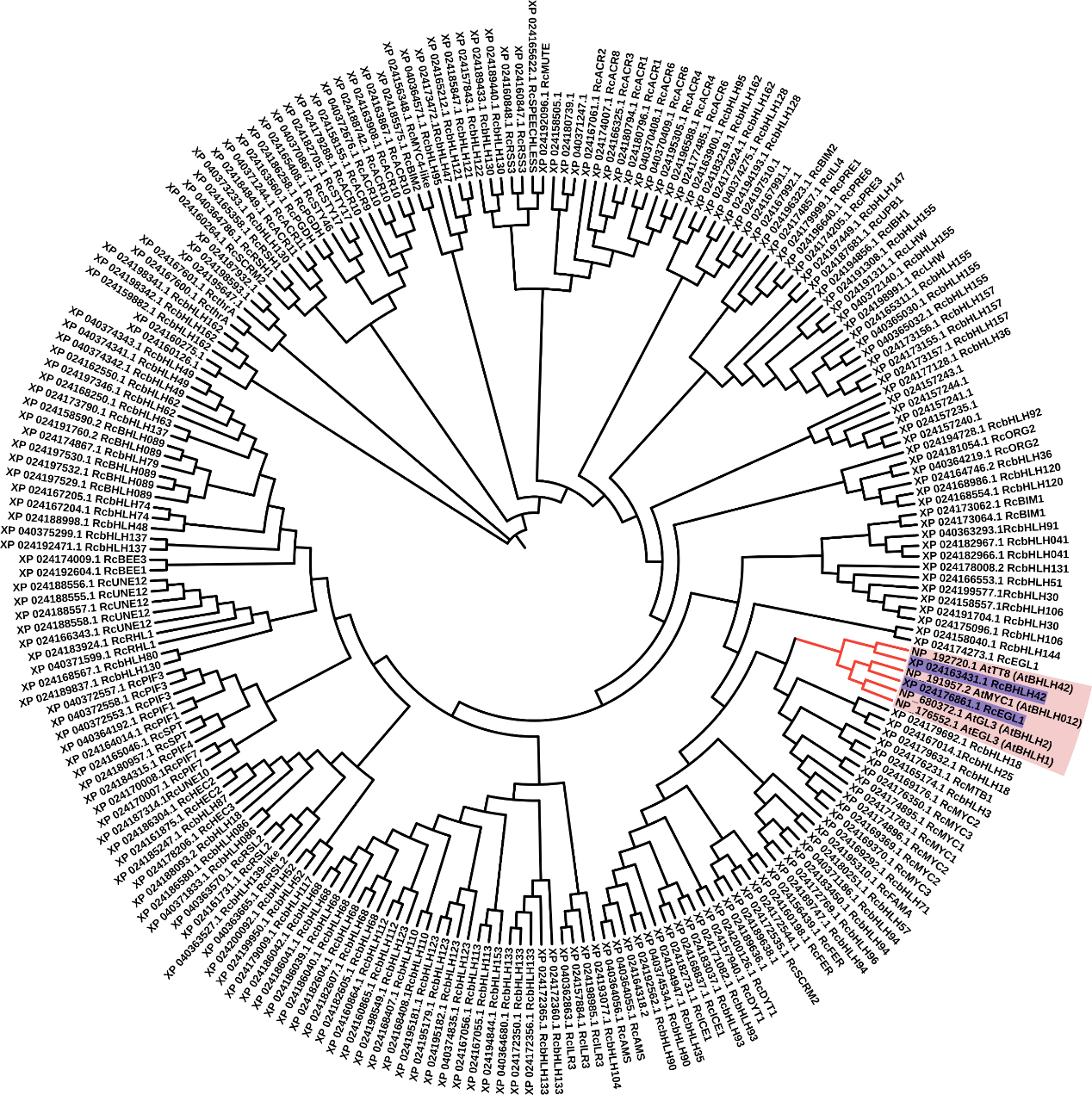


**Supplemental Figure S4.** Phylogenetic tree of rose bHLH family proteins and other bHLH proteins related to anthocyanin biosynthesis in *Arabidopsis.* (AtTT8, NP_192720.2; AtMYC1, NP_191957.2; AtGL3, NP_680372.1; and AtEGL3, NP_974080.1).


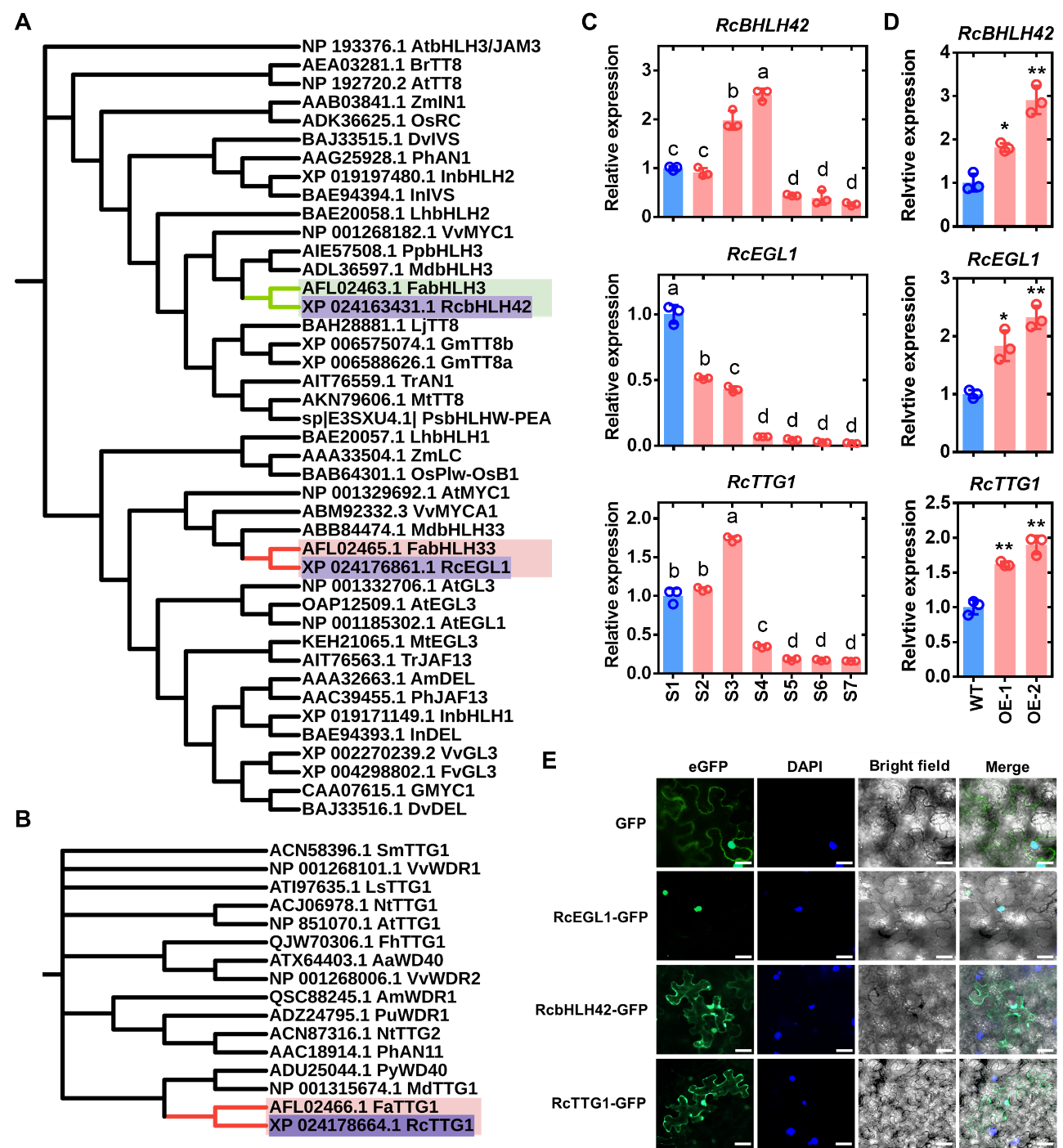


**Supplemental Figure S5.** Analyses of putative components of MBW complexes. **A:** A phylogenetic tree showing the evolutionary relationship between RcBHLH42 and RcEGL1 and bHLH family proteins related to anthocyanin synthesis in other species. **B:** A phylogenetic tree showing the evolutionary relationship between RcTTG1 and WD40 family proteins related to anthocyanin synthesis in other species. **C** and **D**: Relative transcript levels of genes encoding putative components of *RcBHLH42*, *RcEGL1* and *RcTTG1* in rose flowers at different stages of development (C) and in WT and *RcMYB1*-overexpressing lines (OE-1 and OE-2) (D). Values are means ± SDs (n=3). Asterisks indicate significantly different values (Student’s *t* test, * *P* < 0.05 and ** *P* < 0.01). **E:** Subcellular localization of *RcBHLH42*, *RcEGL1* and *RcTTG1* in *N. benthamiana* leaf cells. Nuclei were counterstained with DAPI. The scale bar is 50 μm.


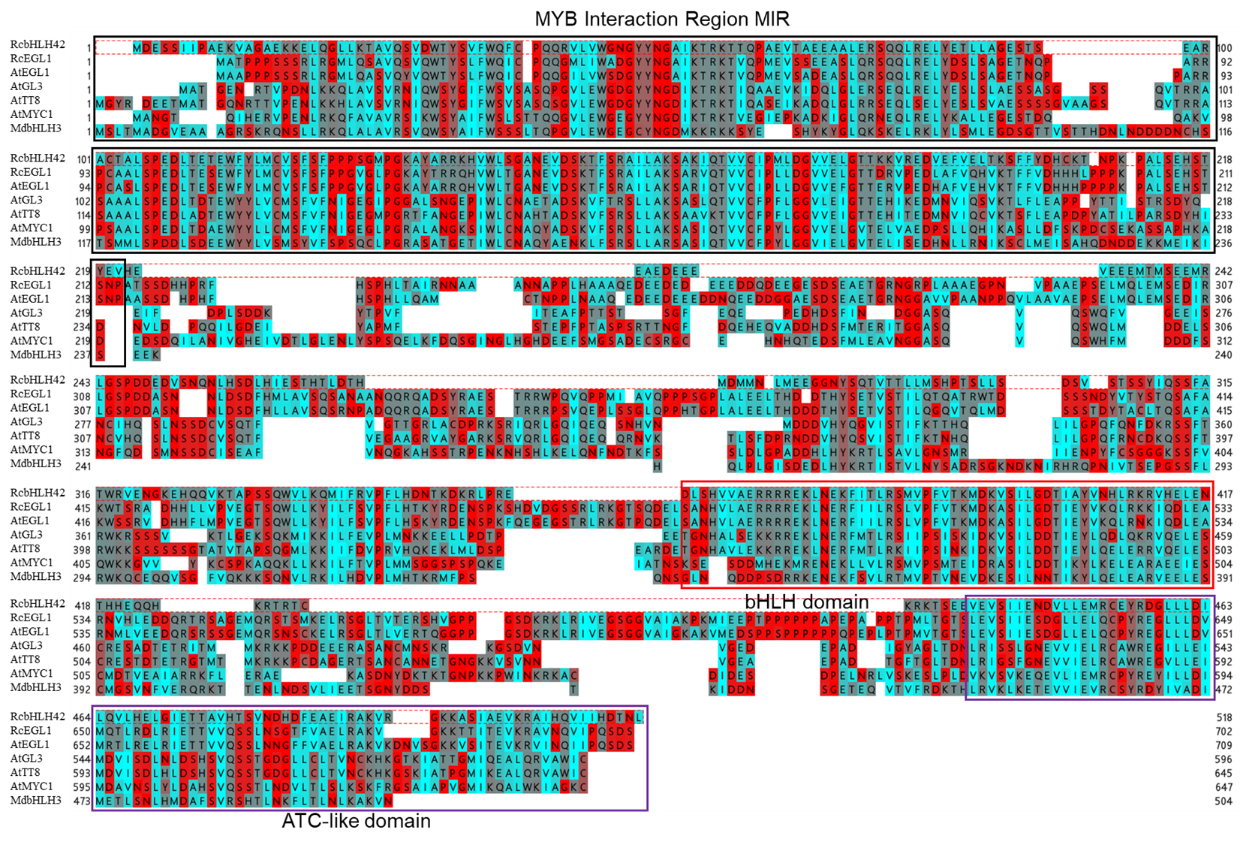


**Supplemental Figure S6.** Multiple sequence alignments of RcbHLH42 and RcEGL1 with sequences from other species. The red box represents the MYB interaction region MIR. The black box represents the bHLH region. The purple box represents the ATC-like domain. *Rc*: *R*. *chinensis* (RcbHLH42, XP_024163431.1; RcEGL1, XP_024176861.1), *Md*: *M*. *domestica* (MdbHLH3, ADL36597.1), *At*: *Arabidopsis thaliana* (AtEGL1, NP_001185302.1; At GL3, NP_001332706.1; AtTT8, NP_192720.2; AtMYC1, NP_001329692.1).


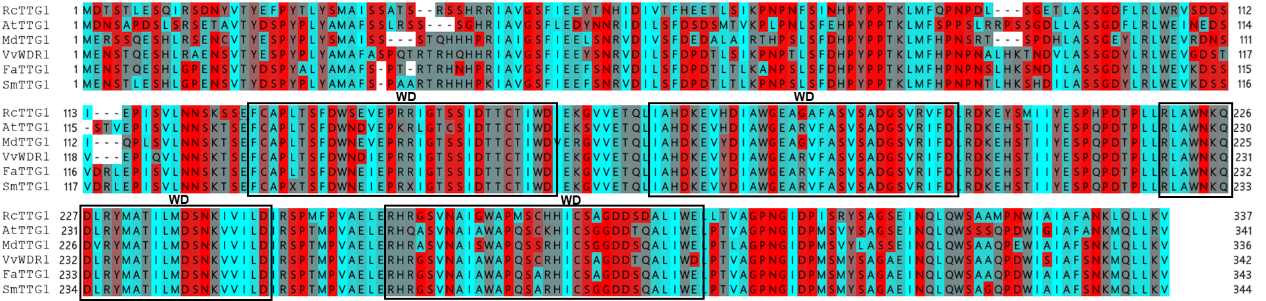


**Supplemental Figure S7.** Multiple sequence alignment of *RcTTG1* with sequences from other species. The black boxes represent the WD motif. *Rc*: *R. chinensis* (RcTTG1, XP_024178664.1), *Md*: *M*. *domestica* (MdTTG1, NP_001315674.1), *Fa*: *Fragaria* × *ananassa* (FaTTG1, AFL02466.1) *Vv*: *Vitis vinifera* (VvWDR1, NP_001268101.1), *At*: *A. thaliana* (AtTTG1, NP_851070.1), *Sm*: *Saussurea medusa* (SmTTG1, ACN58396.1).


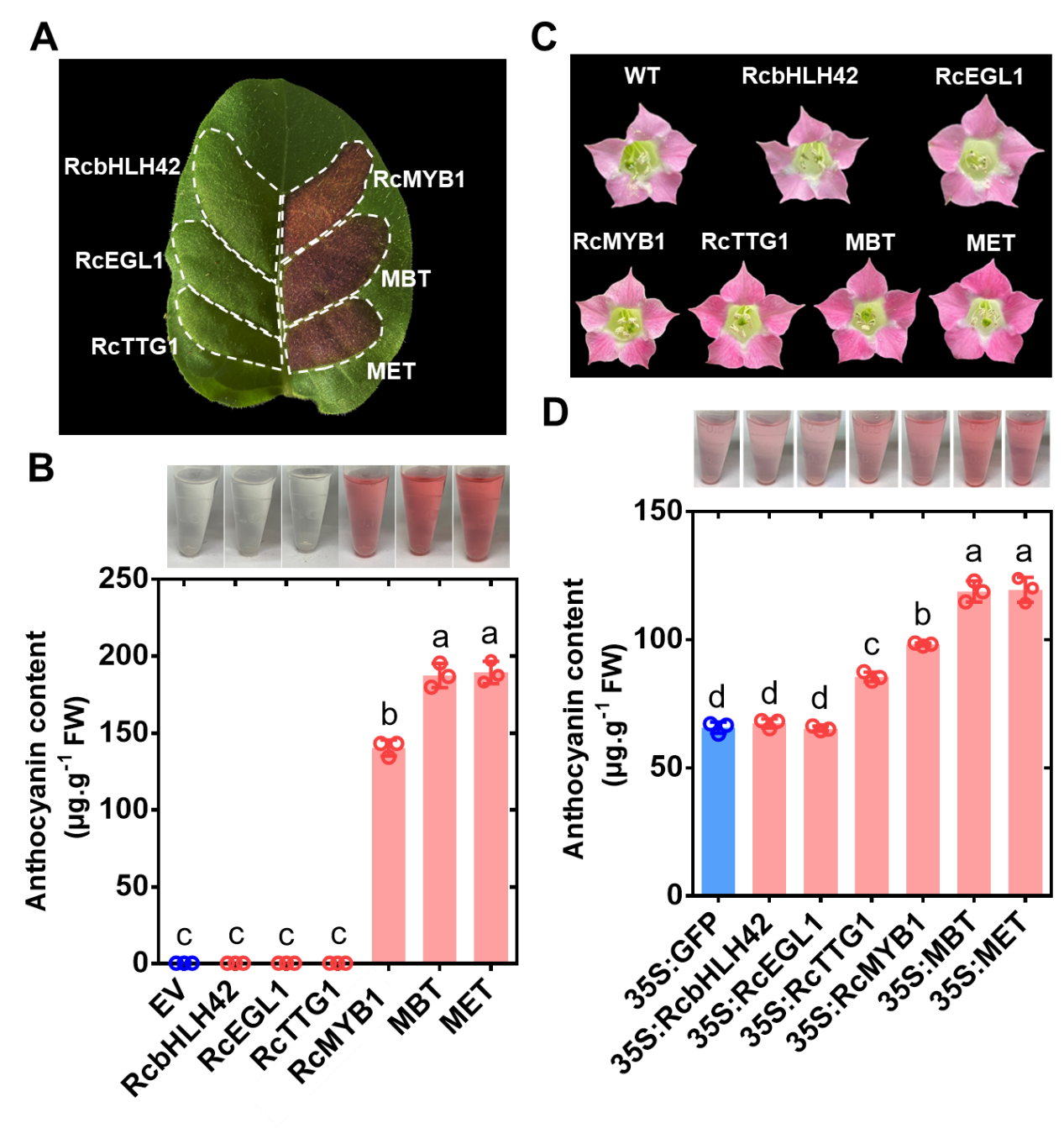


**Supplemental Figure S8.** Transient expression of components of MBW complex alone or in combination. **A**: Tobacco leaf transiently expressing rose genes encoding *RcMYB1*, *RcbHLH42*, *RcEGL1*, *RcTTG1* and two MBW complexes (MBT and MET). **B**: Determination of total anthocyanin content in tobacco leaves. **C**: Phenotypes of WT and transgenic tobacco lines. **D**: Anthocyanin content in WT and transgenic tobacco lines. Values are means ± SDs (n=3). Lowercase letters (a-d) indicate significantly different values (Student’s *t*-test, *P* < 0.05).


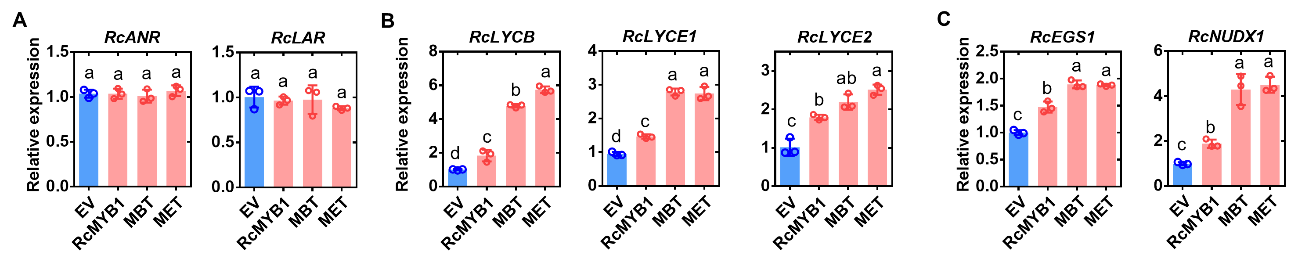


**Supplemental Figure S9.** The expression of *RcANR*, *RcLAR*, *RcLYCB*, *RcLYCE1*, *RcLYCE2*, *RcEGS1* and *RcNUDX1* after transient overexpression of *RcMYB1* and two MBW complexes. **A**: Relative expression of *RcANR* and *RcLAR* after transient overexpression of *RcMYB1* and two MBW complexes. **B**: Relative expression of *RcLYCB*, *RcLYCE1* and *RcLYCE2* after transient overexpression of *RcMYB1* and two MBW complexes. **C**: Relative expression of *RcEGS1* and *RcNUDX1* after transient overexpression of *RcMYB1* and two MBW complexes. Values are means ± SDs (n=3). Lowercase letters (a-d) indicate significantly different values (Student’s *t*-test, *P* < 0.05).


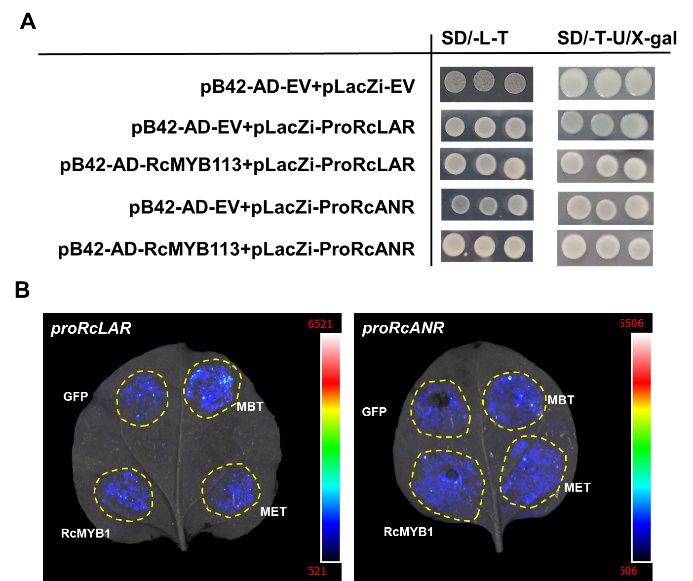


**Supplemental Figure S10.** Two MBW complexes and *RcMYB1* relationships with *RcANR* and *RcLAR*. **A**: Yeast one-hybrid assays showing the binding of *RcMYB1* proteins to the promoters of *RcLAR* and *RcANR*. Yeast cells were grown on selective medium (SD-T-U, SD-Trp-Ura) with 80 mg/L X-gal. **B**: The transient activation test in *N. benthamiana* leaves verified the transcriptional activation ability of *RcMYB1* and two rose MBW complexes (MBT and MET) toward the *RcANR* and *RcLAR*. The luminescence images were captured using a CCD imaging system. Colour in B indicates the interaction strength.


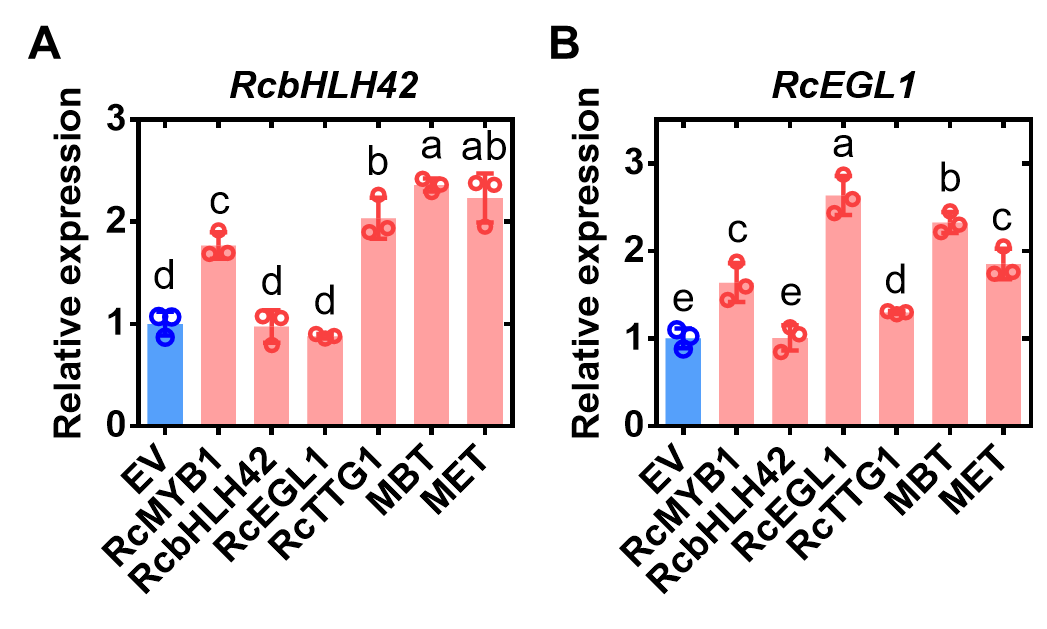


**Supplemental Figure S11.** Relative expression of *RcbHLH42* and *RcEGL1* in transient overexpression of *RcMYB1*, *RcbHLH42*, *RcEGL1* and *RcTTG1* in rose petals. **A:** Relative expression of *RcbHLH42* in transient overexpression of *RcMYB1*, *RcbHLH42*, *RcEGL1*, *RcTTG1* and two MBW complexes (MBT and MET) in rose petals. **B:** Relative expression of *RcEGL1* in transient overexpression of *RcMYB1*, *RcbHLH42*, *RcEGL1*, *RcTTG1* and two MBW complexes (MBT and MET) in rose petals. Values are means ± SDs (n=3). Lowercase letters (a-e) indicate significantly different values (Student’s *t*-test, *P* < 0.05).
