# Supplementary material for: The MYB transcription factor RcMYB1 plays a central role in rose anthocyanin biosynthesis
Source: Hortic Res. 2023 Apr 21;10(6):uhad080. doi: 10.1093/hr/uhad080 (PMC10261888; doi:10.1093/hr/uhad080)
Supplement: Web_Material_uhad080 [file web_material_uhad080.zip › Revised Supplemental Tables.docx]

**Table S1.** Primers used in full length sequence cloning of *RcMYB1*, *RcTTG1*，*RcbHLH42* and *RcEGL1*

| Name | Sequence (5’-3’) | Purposes |
| --- | --- | --- |
| RcMYB1-F | ATGGATGGGGCTTGTAACAA | Full length cloning |
| RcMYB1-R | TCATGTTTTCTCTTCTTCTT |  |
| RcTTG1-F | ATGGAGAACTCGACCCAAGA |  |
| RcTTG1-R | TCAAACCTTCAACAGCTGCA |  |
| RcbHLH42-F | ATGGCTTCACCACCACCTTATAA |  |
| RcbHLH42-R | CTATGATGGAATTATATTCTTCA |  |
| RcEGL1-F | ATGGGTACTAGGCTCCAGAAC |  |
| RcEGL1-R | TCAACAGTTCCTAGCGATTCTCT |  |

**Table S2.** Primers used in qRT-PCR.

| Name | Sequence (5’-3’) | Purposes |
| --- | --- | --- |
| qRcMYB1-F | CCGCTCAACACAAGCATCAC | RT-qPCR |
| qRcMYB1-R | TTCCTCGAACCCCATACGAC |  |
| qRcTTG1-F | CCTCCCACCAAGCTCATGTT |  |
| qRcTTG1-R | CGACGGAGGAGTCTTTGACC |  |
| qRcbHLH42-F | ACGAAGGCAAGGTCTTCTGG |  |
| qRcbHLH42-R | GCCTCTCTTCAGAGTGCTCG |  |
| qRcEGL1-F | TGCCCACTCAGTGCAATCAT |  |
| qRcEGL1-R | GGTGCAATTGCTGATCCACG |  |
| qRcCHSab-F | CGAGATATCACAATGGTGACCGT |  |
| qRcCHSab-R | ACATGCGCTGGAATTTCTCCTTGA |  |
| qRcCHSc-F | CCCAATATCGAAATGGTGACTGTCG |  |
| qRcCHSc-R | TGGAATTTCTTCTTGAGCTCAACCT |  |
| qRcCHI-F | CGCCGTTAAGTGGAAGGGT |  |
| qRcCHI-R | TGGCAGTATCATCGTCACCTG |  |
| qRcF3H-F： | TTCAAGAACGCCGATCACCA |  |
| qRcF3H-R： | TGGCTCCTCCAGAATAGGCT |  |
| qRcF3'H-F： | CTGAAACCTTCAACACACTCGACTC |  |
| qRcF3'H-R： | ATTTGCCGGAGAAAAGAAGCCG |  |
| qRcDFR-F： | CGTAGGTTCATGGCTCGTCA |  |
| qRcDFR-R： | TCCACAGCGTCAAGTGAGTC |  |
| qRcANS-F | AGCTCATGGAACGGGTCAAG |  |
| qRcANS-R | TTGCCCGGAAGCATTGTTTG |  |
| qRcUFGT-F | GAGCCACAAAGTTGCTAGTTCTAG |  |
| qRcUFGT-R | CTTTTTGCCGGAACTCGAGAAG |  |
| qRcGT1-F | CCTCGACATGTTTTGCGACG |  |
| qRcGT1-R | ACAGACTGTTGGTTGTGCGA |  |
| qRc3GT5-F | GAAGTAGGGAAGGTGATGAGAAGC |  |
| qRc3GT5-R | TCAAATTGGCGCAGCTAGATC |  |

**Table S3.** Primers used in vectors construction.

| Name | Sequence (5’-3’) | Purposes |
| --- | --- | --- |
| 2300-RcMYB1-F | ttggagaggacagggtacccgggATGGATGGGGCTTGTAACAACA | Overexpression and Subcellular localization |
| 2300-RcMYB1-R | ccatggtactagtgtcgactctagaTGTTTTCTCTTCTTCTTTTGGATGA |  |
| 2300-RcRcTTG1-F | ttggagaggacagggtacccgggATGGAGAACTCGACCCAAGA |  |
| 2300-RcRcTTG1-R | ccatggtactagtgtcgactctagaAACCTTCAACAGCTGCATCT |  |
| 2300-RcBHLH42-F | ttggagaggacagggtacccgggATGGCTACACCGCCACCG |  |
| 2300-RcBHLH42-R | ccatggtactagtgtcgactctagaAGAGTCAGATTGGGGTATCACT |  |
| 2300-RcEGL1-F | ttggagaggacagggtacccgggATGGCCAATGGGACTCAAATC |  |
| 2300-RcEGL1-R | ccatggtactagtgtcgactctagaACACTTACCAGCAATTTTCCA |  |
| AD-RcMYB1-F | atggccatggaggccagtgaattcATGGATGGGGCTTGTAACAA |  |
| AD-RcMYB1-R | ctgcagctcgagctcgatggatccTCATGTTTTCTCTTCTTCTT |  |
| AD-RcTTG1-F | atggccatggaggccagtgaattcATGGAGAACTCGACCCAAGA | Y2H assay |
| AD-RcTTG1-R | ctgcagctcgagctcgatggatccTCAAACCTTCAACAGCTGCA |  |
| BD-RcBHLH42-F | catatggccatggaggccgaattcATGGCTTCACCACCACCTTA |  |
| BD-RcBHLH42-R | cggccgctgcaggtcgacggatccCTATGATGGAATTATATTCT |  |
| BD-RcTTG1-F | catatggccatggaggccgaattcATGGAGAACTCGACCCAAGA |  |
| BD-RcTTG1-R | cggccgctgcaggtcgacggatccTCAAACCTTCAACAGCTGCA |  |
| BD-RcEGL1-F | catatggccatggaggccgaattcATGGCCAATGGGACTCAA |  |
| BD-RcEGL1-R | cggccgctgcaggtcgacggatccTCAACACTTACCAGCAATTT |  |
| PXY106-RcMYB1-F | atcgaggacgccggcggatccATGGATGGGGCTTGTAACAACA |  |
| PXY106-RcMYB1-R | acgaaagctctgcaggtcgacTCATGTTTTCTCTTCTTCTTTTGGA |  |
| PXY106-RcTTG1-F | atcgaggacgccggcggatccATGGAGAACTCGACCCAAGA | Bimolecular Fluorescent Complementation (BiFC) analysis |
| PXY106-RcTTG1-R | acgaaagctctgcaggtcgacTCAAACCTTCAACAGCTGCA |  |
| PXY104-RcBHLH42-F | attacaggtacccggggatccATGGCTACACCGCCACCGAG |  |
| PXY104-RcBHLH42-R | cacgctgccaccgccgtcgacAGAGTCAGATTGGGGTATCA |  |
| PXY104-RcTTG1-F | attacaggtacccggggatccATGGAGAACTCGACCCAAGA |  |
| PXY104-RcTTG1-R | cacgctgccaccgccgtcgacAACCTTCAACAGCTGCATCT |  |
| PXY104-RcEGL1-F | attacaggtacccggggatccATGGCCAATGGGACTCAAAT |  |
| PXY104-RcEGL1-R | cacgctgccaccgccgtcgacACACTTACCAGCAATTTTCC |  |
| PEG104-3*FLAG-RcMYB1-F | aaaaaagcaggctcaggggatatcATGGATGGGGCTTGTAACAACA |  |
| PEG104-3*FLAG-RcMYB1-R | accgttaattaacccgctgatatcTGTTTTCTCTTCTTCTTTTGGATGA |  |
| PEG104-3*FLAG-RcTTG1-F | aaaaaagcaggctcaggggatatcATGGAGAACTCGACCCAAGA | Co-IP assay |
| PEG104-3*FLAG-RcTTG1-R | accgttaattaacccgctgatatcAACCTTCAACAGCTGCATCT |  |
| PEG104-Myc-RcBHLH42-F | aaaaaagcaggctcaggggatatcATGGCTACACCGCCACCG |  |
| PEG104-Myc-RcBHLH42-R | accgttaattaacccgctgatatcAGAGTCAGATTGGGGTATCACT |  |
| PEG104-Myc-RcTTG1-F | aaaaaagcaggctcaggggatatcATGGAGAACTCGACCCAAGA |  |
| PEG104-Myc-RcTTG1-R | accgttaattaacccgctgatatcAACCTTCAACAGCTGCATCT |  |
| PEG104-Myc-RcEGL1-F | aaaaaagcaggctcaggggatatcATGGCCAATGGGACTCAAATC |  |
| PEG104-Myc-RcEGL1-R | accgttaattaacccgctgatatcACACTTACCAGCAATTTTCCA |  |
| PEG104-Myc-RcMYB1-F | aaaaaagcaggctcaggggatatcATGGATGGGGCTTGTAACAACA |  |
| PEG104-Myc-RcMYB1-R | accgttaattaacccgctgatatcTGTTTTCTCTTCTTCTTTTGGATGA |  |
| pB42AD-RcMYB1-F | ccagattatgcctctcccgaattcATGGATGGGGCTTGTAACAA |  |
| pB42AD-RcMYB1-R | cgaagaagtccaaagcttctcgagTCATGTTTTCTCTTCTTCTT |  |
| pIacZ-proRcCHSab-F | cctttgatattggatcggaattcGCTGCGATCTGGGCAATTGAAAC | Yeast one-hybrid assay |
| pIacZ-proRcCHSab-R | cccgggtaccgagctcgaattcGGCTTCGAAGTTGGAAAACATCCTG |  |
| pIacZ-proRcCHSc-F | cctttgatattggatcggaattcGCAATGGCTCATGATCCTACTGT |  |
| pIacZ-proRcCHSc-R | cccgggtaccgagctcgaattcCCTCAAGGGGCCATTTCTCTATCA |  |
| pIacZ-proRcCHI-F | cctttgatattggatcggaattcCTCACCACCAGCATCAGAGTCATAA |  |
| pIacZ-proRcCHI-R | cccgggtaccgagctcgaattcGTTTTGTCGGAGTAGCAAGAGAACC |  |
| pIacZ-proRcF3H-F | cctttgatattggatcggaattcTGCGCTAGTCCTGTTAGGTGAATAT |  |
| pIacZ-proRcF3H-R | cccgggtaccgagctcgaattcGTCCCGGATGAACTTGTCCCAATT |  |
| pIacZ-proRcF3'H-F | cctttgatattggatcggaattcATAAGCAGTATGCAATGCTTCACGT |  |
| pIacZ-proRcF3'H-R | cccgggtaccgagctcgaattcCTTTACCCGAGAGGGAGAGCATC |  |
| pIacZ-proRcDFR-F | cctttgatattggatcggaattcGCTTAATCAAATCCTTGACCGATTC |  |
| pIacZ-proRcDFR-R | cccgggtaccgagctcgaattcATAGGTGAAACTATGTGTGCTTTGC |  |
| pIacZ-proRcANS-F | cctttgatattggatcggaattcGTCTCTGGTGATGGCAGAGGAAGA |  |
| pIacZ-proRcANS-R | cccgggtaccgagctcgaattcGTCTCACGTACTCCTTTGGGATCG |  |
| pIacZ-proRcUFGT-F | cctttgatattggatcggaattcAAGCATAGCATAATCGGGTTATACG |  |
| pIacZ-proRcUFGT-R | cccgggtaccgagctcgaattcCTAGCAACTTTGTGGCTCATTATG |  |
| pIacZ-proRcGT1a-F | cctttgatattggatcggaattcCCAACCAGGTCTTTGAGGTTAAACT |  |
| pIacZ-proRcGT1a-R | cccgggtaccgagctcgaattcACCATGGAAATTAGGTGGCCTAGTC |  |
| pIacZ-proRc3GT5-F | cctttgatattggatcggaattcCCTACGACAGACCTGGTATCATAAC |  |
| pIacZ-proRc3GT5-R | cccgggtaccgagctcgaattcGTACGCGGTACGCTAGGATATATTAGT |  |
| pIacZ-proRcMYB1-F | cctttgatattggatcggaattcTAAGTTGCGAATGACTCGTTATGAG |  |
| pIacZ-proRcMYB1-R | cccgggtaccgagctcgaattcGATCATCTTCCTCTCTAGTCCAAGC |  |
| pIacZ-proRcLYCE-1-F | cctttgatattggatcggaattcGGACTTACCAGCAAGGCAGCAAATC |  |
| pIacZ-proRcLYCE-1-R | cccgggtaccgagctcgaattcCAACGCAGTCCATGACCACCAAAAC |  |
| pIacZ-proRcLYCE-2-F | cctttgatattggatcggaattcGCATTAATGGGAAATATCTATTAGCAATCTGG |  |
| pIacZ-proRcLYCE-2-R | cccgggtaccgagctcgaattcCCATGAAATTCCAATATGTCTGACAATTCC |  |
| pIacZ-proRcLYCB-1-F | cctttgatattggatcggaattcGGGATGAATGACTGCTTCCTCAACT |  |
| pIacZ-proRcLYCB-1-R | cccgggtaccgagctcgaattcGCTGACAAAGGTGTCCATTATAGAGTGG |  |
| pIacZ-proRcANR-F | cctttgatattggatcggaattcGTCGAGAAGACCATGTTTGAAGCAT |  |
| pIacZ-proRcANR-R | cccgggtaccgagctcgaattcGTCGAATCGTACTGTGACAGAGCA |  |
| pIacZ-proRcLAR-F | cctttgatattggatcggaattcTCCCCAATACAACATGCTTGCGTA |  |
| pIacZ-proRcLAR-R | cccgggtaccgagctcgaattcTGGTTGCTTGGTTCACCAACATAA |  |
| pIacZ-proRcEGS1-F | cctttgatattggatcggaattcAAATGGAGTCTACCCCTTACGGGTA |  |
| pIacZ-proRcEGS1-R | cccgggtaccgagctcgaattcTGGGATTATACACTCTGTGATGCAA |  |
| pIacZ-proRcNUDX1-F | cctttgatattggatcggaattcGGATGTAGGAGTGATCAAGTCAATG |  |
| pIacZ-proRcNUDX1-R | cccgggtaccgagctcgaattcGCTCTCTCCTGAAACGTTTGTAAAG |  |
| Luc-proRcCHSab-F | gtcgacggtatcgataagcttGCTGCGATCTGGGCAATTGAAAC |  |
| Luc-proRcCHSab-R | cgctctagaactagtggatccGGCTTCGAAGTTGGAAAACATCCTG |  |
| Luc-proRcCHSc-F | gtcgacggtatcgataagcttGCAATGGCTCATGATCCTACTGT | Dual-luciferase reporter assay |
| Luc-proRcCHSc-R | cgctctagaactagtggatccCCTCAAGGGGCCATTTCTCTATCA |  |
| Luc-proRcCHI-F | gtcgacggtatcgataagcttCTCACCACCAGCATCAGAGTCATAA |  |
| Luc-proRcCHI-R | cgctctagaactagtggatccGTTTTGTCGGAGTAGCAAGAGAACC |  |
| Luc-proRcF3H-F | gtcgacggtatcgataagcttTGCGCTAGTCCTGTTAGGTGAATAT |  |
| Luc-proRcF3H-R | cgctctagaactagtggatccGTCCCGGATGAACTTGTCCCAATT |  |
| Luc-proRcF3'H-F | gtcgacggtatcgataagcttATAAGCAGTATGCAATGCTTCACGT |  |
| Luc-proRcF3'H-R | cgctctagaactagtggatccCTTTACCCGAGAGGGAGAGCATC |  |
| Luc-proRcDFR-F | gtcgacggtatcgataagcttGCTTAATCAAATCCTTGACCGATTC |  |
| Luc-proRcDFR-R | cgctctagaactagtggatccATAGGTGAAACTATGTGTGCTTTGC |  |
| Luc-proRcANS-F | gtcgacggtatcgataagcttGTCTCTGGTGATGGCAGAGGAAGA |  |
| Luc-proRcANS-R | cgctctagaactagtggatccGTCTCACGTACTCCTTTGGGATCG |  |
| Luc-proRcUFGT-F | gtcgacggtatcgataagcttAAGCATAGCATAATCGGGTTATACG |  |
| Luc-proRcUFGT-R | cgctctagaactagtggatccCTAGCAACTTTGTGGCTCATTATG |  |
| Luc-proRcGT1a-F | gtcgacggtatcgataagcttCCAACCAGGTCTTTGAGGTTAAACT |  |
| Luc-proRcGT1a-R | cgctctagaactagtggatccACCATGGAAATTAGGTGGCCTAGTC |  |
| Luc-proRc3GT5-F | gtcgacggtatcgataagcttCCTACGACAGACCTGGTATCATAAC |  |
| Luc-proRc3GT5-R | cgctctagaactagtggatccGTACGCGGTACGCTAGGATATATTAGT |  |
| Luc-proRcMYB1-F | gtcgacggtatcgataagcttTAAGTTGCGAATGACTCGTTATGAG |  |
| Luc-proRcMYB1-R | cgctctagaactagtggatccGATCATCTTCCTCTCTAGTCCAAGC |  |
| Luc-proRcLYCE-1-F | gtcgacggtatcgataagcttGGACTTACCAGCAAGGCAGCAA |  |
| Luc-proRcLYCE-1-R | cgctctagaactagtggatccCAACGCAGTCCATGACCACCAAAAC |  |
| Luc-proRcLYCE-2-F | gtcgacggtatcgataagcttTCAATCACTGATTGATCAGAAGCTC |  |
| Luc-proRcLYCE-2-R | cgctctagaactagtggatccCCATGAAATTCCAATATGTCTGACA |  |
| Luc-proRcLYCB-1-F | gtcgacggtatcgataagcttGGGATGAATGACTGCTTCCTCAACT |  |
| Luc-proRcLYCB-1-R | cgctctagaactagtggatccGCTGACAAAGGTGTCCATTATAGAGTGG |  |
| Luc-proRcANR-F | gtcgacggtatcgataagcttGTCGAGAAGACCATGTTTGAAGCAT |  |
| Luc-proRcANR-R | cgctctagaactagtggatccGTCGAATCGTACTGTGACAGAGCA |  |
| Luc-proRcLAR-F | gtcgacggtatcgataagcttTCCCCAATACAACATGCTTGCGTA |  |
| Luc-proRcLAR-R | cgctctagaactagtggatccTGGTTGCTTGGTTCACCAACATAA |  |
| Luc-proRcEGS1-F | gtcgacggtatcgataagcttAAATGGAGTCTACCCCTTACGGGTA |  |
| Luc-proRcEGS1-R | cgctctagaactagtggatccTGGGATTATACACTCTGTGATGCAA |  |
| Luc-proRcNUDX1-F | gtcgacggtatcgataagcttGGATGTAGGAGTGATCAAGTCAATG |  |
| Luc-proRcNUDX1-R | cgctctagaactagtggatccGCTCTCTCCTGAAACGTTTGTAAAG |  |
| ChIP-RcMYB1-F1 | GGTCTCACATACATGCAGTTGTCTG | ChIP-qPCR |
| ChIP-RcMYB1-R1 | CAGTAGAAGATGAATTTTTCAGATA |  |
| ChIP-RcMYB1-F2 | AAAGAAGAAACAGCTTTCTGCTAA |  |
| ChIP-RcMYB1-R2 | TGGTTTGAGGCACATGTTGCATAG |  |
| ChIP-RcMYB1-F3 | ACACACACACACGAGGTTATATATA |  |
| ChIP-RcMYB1-R3 | TGTTATGATTATGGAGTCACTTAGC |  |
| ChIP-RcMYB1-F4 | TCCCCCTGCTGCATTGTATACATG |  |
| ChIP-RcMYB1-R4 | ACTGCAGCAATGTACGTGTTCCTAC |  |
| ChIP-RcLYCB-F1 | CAGACCGAATGACTGAAATTACGTC |  |
| ChIP-RcLYCB-R1 | CAGAGGTTGACATTTTAACGACTTC |  |
| ChIP-RcLYCB-F2 | TGGATCCGATGATACCAAAGCAAAG |  |
| ChIP-RcLYCB-R2 | TTTGGTAGTGCAGCATTTGTTTGAG |  |
| ChIP-RcLYCE-1-F1 | AAGTACAGTGAGAGCTGCAGTGCTA |  |
| ChIP-RcLYCE-1-R1 | TATACACAGCGGTGCAAGTCCAC |  |
| ChIP-RcLYCE-1-F2 | CTATTGGACTGGGCGTGGAGATG |  |
| ChIP-RcLYCE-1-R2 | CGCTGAGTTAAGGGCCTCTGTCA |  |
| ChIP-RcLYCE-2-F1 | GGCATCCTATGTGAATTCATCTGAT |  |
| ChIP-RcLYCE-2-R1 | GCTGTTGTCAGAATTTGATTGGCCT |  |
| ChIP-RcLYCE-2-F2 | CAGTGCCGTACTGCTTTCATTCC |  |
| ChIP-RcLYCE-2-R2 | AACATCACATGCTTGCTAGAGAACC |  |
| ChIP-RcNUDX1-F1 | CTCCACGTGTAGGGCTGAGATTGT |  |
| ChIP-RcNUDX1-R1 | GAACGCCCTCATGTCACAGTTACGA |  |
| ChIP-RcNUDX1-F2 | GAAAAGTCCTTGCACTCTCCAATCG |  |
| ChIP-RcNUDX1-R2 | GTTGCCTAGCGATGGGTAGTTTG |  |
| ChIP-RcEGS1-F1 | TGGGACCAGAATGGGTTTCATGATC |  |
| ChIP-RcEGS1-R1 | AATTTGCACTCTCTGTGAGGCAAGA |  |
| ChIP-RcEGS1-F2 | GTGACATCGCCGGAAATCTCACCA |  |
| ChIP-RcEGS1-R2 | GTGAGATTTCCGGCAATCTCCGGC |  |
